# Organelle-targeted precision cancer theranostics: From molecular design to NIR-II imaging

**DOI:** 10.7150/thno.137525

**Published:** 2026-09-18

**Authors:** Dandan Chen, Beiling Zheng, Zhixiang Lu, Yaohui He, Pengfei Lyu

**Affiliations:** 1 Department of Breast and Thyroid Surgery, The Affiliated Nanhua Hospital, Hengyang Medical School, University of South China, Hengyang, 421001, China.; 2 Institute for Future Sciences, MOE Key Laboratory of Rare Pediatric Diseases, Department of Cell Biology and Genetics, School of Basic Medical Sciences, Hengyang Medical School, University of South China, Hengyang, 421001, China.; 3 State Key Laboratory of Vaccines for Infectious Diseases, Xiang' An Biomedicine Laboratory, Fujian Engineering Research Center of Molecular Theranostic Technology, Fujian Provincial Key Laboratory of Innovative Drug Target Research, School of Pharmaceutical Sciences, Xiamen University, Xiamen, 361102, China.; 4 Sichuan Clinical Research Center for Radiation and Therapy, The second Affiliated Hospital of Chengdu Medical College, Nuclear Industry 416 Hospital, Chengdu, 610051, China.; 5 Department of Breast Surgery, The First Affiliated Hospital of Hainan Medical University, Haikou, 570102, China.

**Keywords:** fluorescence imaging, organelle-targeted therapy, theranostics, NIR-II imaging, cancer nanomedicine

## Abstract

The disruption of organelle homeostasis is a hallmark in the process of tumor occurrence and development. The abnormalities of these subcellular structures not only indicate the pathological state of the body tissue, but also provide actionable targets for clinical intervention. In recent years, organelle targeted fluorescence imaging technology has shown unique advantages in tumor identification, subcellular localization and dynamic function monitoring.

In this review, we systematically summarize the latest progress of organelle-targeted theranostics platforms for precise cancer diagnosis and treatment, focusing on five major organelles: Mitochondria (Mt), Lysosomes, Endoplasmic reticulum (ER), Nucleus and Golgi apparatus (GA). We discussed the molecular design strategies for these platforms to achieve targeted organelle diagnosis imaging. At the same time, we focused on comparing the activation mechanism of targeting motifs, imaging platforms, and advanced strategies such as Aggregation-Induced Emission (AIE), ratiometric imaging and signal amplification. These common strategies jointly ienhance the accuracy of tumor diagnosis. In addition, this review explores how organelle imaging information has evolved from a simple localization imaging tool to the basis for tumor treatment decision-making, including determining the best treatment window, regulating probe activation, monitoring intracellular transport, evaluating organelle damage, multi-organelle cascade therapies, and promoting immunogenic cell death. In addition to these cellular strategies, the emergence of Near-infrared II (NIR-II) fluorescence imaging provides the latest and key technical advantages for deep tissue tracking of biological distribution, tumor retention and treatment timing *in vivo*. This review aims to overcome the technical challenges of insufficient tissue penetration depth and insufficient targeted specificity in the field of tumor diagnosis and treatment, so as to promote the development of high-efficiency, low-toxic and accurate tumor platforms and accelerate their clinical translation.

## 1. Introduction

Cancer development is a complex, multi-step process driven by the gradual accumulation of carcinogenic mutations and the continuous exposure of multiple carcinogenic factors. Cancer cells are characterized by uncontrolled proliferation, high invasiveness, metastatic potential, profound heterogeneity and disordered tumor microenvironment. Epidemiological data show that approximately 18.5 million new cases and 10.4 million deaths were reported in 2023. By 2050, the global cancer burden is expected to rise to 30.5 million new cases and 18.6 million deaths, an increase of 60.7% and 74.5 % respectively compared with the recent baseline estimates. Therefore, cancer remains one of the leading causes of global morbidity and mortality, and its disease burden is expected to escalate further 1. Currently, surgery, radiotherapy, and chemotherapy constitute the mainstays of clinical cancer management. However, the surgical treatment of tumor infiltration or metastatic lesions remains highly challenging; radiotherapy can cause collateral damage to healthy tissues surrounding the lesion; and chemotherapy is often limited by low selectivity, dose-limited toxicity and acquired drug resistance 2, 3. These limitations have spurred the development of precision cancer diagnosis and therapy. The goal of precision oncology is to devise strategies to improve the accuracy of tumor diagnosis and treatment controllability by identifying tumor-specific biological abnormalities. Tumor-associated alterations are not confined to the cell surface or extracellular tumor microenvironment. In the process of malignant transformation and tumor progression, key intracellular organelles—including Mitochondria (Mt), Lysosomes, Endoplasmic reticulum (ER), Nucleus, and Golgi apparatus (GA), undergo marked changes in their structure, function, and microenvironment 4-6. For example, Mitochondrial Membrane Potential (ΔΨm) and metabolic changes, lysosomal acidification and enzyme activity changes, endoplasmic reticulum stress (ERs), nuclear transport and DNA homeostasis abnormalities 7, and changes in GA trafficking or glycosylation 8, collectively contribute to tumor growth, invasion, therapeutic resistance, and immune regulation. Simultaneously, these abnormalities provide sources of diagnostic contrast and reveal subcellular structural targets that can be used for therapeutic intervention 9.

Fluorescence imaging is ideally suited for monitoring these dynamic processes. By integrating optical signals with targeting units or responsive mechanisms, fluorescent probes enable the visualization of tumor-associated molecular features or microenvironmental changes through alterations in fluorescence signals. When combined with organelle-targeting strategies, these imaging platforms can not only reveal their subcellular distribution but also enhance the accuracy of tumor diagnosis by exploiting the unique physiological and pathological characteristics of specific organelles in tumor cells. This imaging strategy facilitates the distinction between tumors and normal tissues, assessment of cellular functional states, delineation of tumor margins, assistance in surgical resection, and tracking of disease progression 10.

In the integrated strategy of precision cancer diagnosis and therapy, the role of fluorescent signals extends far beyond simple tumor visualization. They can be utilized to identify optimal treatment windows, optimize drug dosages and sequences, distinguish active drugs from inactive precursors, and track the intracellular transport of drugs 11. In addition, they can provide early indications of organelle damage and therapeutic efficacy.

Accordingly, this review first analyzes organelle-targeted fluorescence imaging platforms used for tumor diagnosis and categorizes them based on their targeting motifs. Our focus encompasses not only the accumulation of imaging platforms within organelles but also their ultimate tumor selectivity, intracellular delivery efficiency, fluorescence reliability, and biosafety.

Moreover, organelle damage not only determines local therapeutic outcomes but may also elicit broader biological responses. Mitochondrial dysfunction, lysosomal membrane permeabilization, ERs, and nuclear DNA damage can activate distinct yet interconnected cell death pathways 12. By regulating intracellular transport or simultaneously targeting multiple organelles, these damage signals can be amplified to enhance therapeutic efficacy. Furthermore, a variety of treatment modalities—including Photodynamic Therapy (PDT), Photothermal Therapy (PTT), catalytic therapy, chemotherapy, and Immunotherapy (IMT)—can be combined to overcome challenges such as tumor hypoxia, antioxidant defense mechanisms, and the restricted diffusion range of Reactive Oxygen Species (ROS) 13.

Near-infrared II (NIR-II) fluorescence imaging can overcome the limited penetration depth of traditional optical imaging. Its reduced tissue scattering and low autofluorescence allow long-term monitoring of probe biodistribution, tumor accumulation, and therapeutic responses *in vivo* 14. Nevertheless, NIR-II primarily provides tissue-level information and currently cannot directly resolve organelle localization or function in deep tissues 15. Therefore, in integrated studies of organelle-targeted diagnosis and therapy, NIR-II imaging is primarily employed to evaluate *in vivo* pharmacokinetic behaviors, tumor-targeted accumulation, and therapeutic time windows, whereas cellular microscopy and functional biological assays are further utilized to validate subcellular localization, organelle damage mechanisms, and therapeutic responses 16.

This review systematically summarizes recent advances spanning the design of organelle-targeted imaging platforms to integrated theranostic applications, and comprehensively analyzes the role of organelle targeting strategies in precision cancer diagnosis and therapy. First, this review focuses on five key organelles—Mt, Lysosomes, ER, Nucleus, and GA—and discusses multi-level organelle-targeting design strategies, including functional motif-mediated organelle anchoring mechanisms, tumor microenvironment-responsive activation, and fluorescence signal regulation strategies. Subsequently, the application value of organelle imaging information in cancer treatment is further evaluated, covering optimization of therapeutic time windows, intracellular trafficking tracking, organelle-directed phototherapy, multimodal combination therapy, regulation of cell death pathways, and activation of antitumor immunity. Additionally, this review discusses the potential of NIR-II fluorescence imaging in enhancing *in vivo* cancer theranostic capabilities and summarizes the key challenges that must be addressed for the clinical translation of organelle-targeted probes, including biosafety, pharmacokinetic evaluation, multiscale imaging validation, and the establishment of imaging-guided therapeutic decision-making systems (**Scheme 1**).

## 2. Design principles of organelle-targeted fluorescence imaging platforms

### 2.1 Organelle-targeted tumor diagnostic strategies

Abnormalities in organelle structure and function provide an important biological basis for the diagnosis of tumor cells at the subcellular level. The core principle of organelle-targeted imaging is to exploit the unique biochemical environments or molecular recognition sites of different subcellular structures, enabling fluorescent probes to selectively accumulate within specific organelles and produce significant fluorescence 23. Unlike conventional strategies that rely on the enhanced permeability and retention (EPR) effect for passive tumor accumulation, organelle-targeted platforms incorporate functional targeting motifs that allow probes to recognize specific intracellular structures. This strategy has significantly improved the accuracy of subcellular localization and tumor imaging. And the distinct membrane properties, ionic environments, protein expression patterns, and metabolic states of different organelles provide a variety of recognition mechanisms for targeted delivery 24.

The existing fluorescent imaging platforms typically includes three functional modules: (i) an organelle-targeting moiety, which facilitates the entry or retention of the imaging platform within the subcellular structure; (ii) the tumor recognition or activation module, which responds to specific enzyme molecules, metabolites, reactive species or local physical properties; (iii) a fluorescent reporter unit, which can enable persistent localization, activatable signal recovery, ratiometric quantification or dynamic functional imaging. Although the always-on probe facilitates the mapping of, its fluorescent signals are easily susceptible to background interference and concentration variability. In contrast, the activatable probe only releases significant fluorescence upon recognition of specific disease-associated features, whereas the ratiometric probe mainly improves quantitative accuracy 25. Therefore, the choice of targeting motifs not only determines the subcellular distribution of the probe but also critically influences tumor selectivity, imaging signal-to-noise ratio, and therapeutic responses. The design of integrated organelle-targeted diagnostic and therapeutic platforms also requires a comprehensive evaluation of targeting efficiency, intracellular stability, controllable responsiveness and biological safety.

Based on these design principles, different organelle-targeting strategies exhibit distinct characteristics (**Table 1**). Mitochondrial and Lysosomal targeting systems benefit from a mature design framework based on relatively stable physicochemical properties but remain vulnerable to interference from similar environments in normal tissues. In contrast, the targeting strategies for the GA, ER, and Nucleus mainly rely on molecular recognition mechanisms, offering higher localization selectivity. However, their functional motifs remain limited, and their *in vivo* application is still constrained by delivery efficiency and biosafety. In the future, the development of organelle-targeted imaging platforms not only requires enhanced subcellular localization but also improved integration with tumor-specific responses, intelligent activation mechanisms, and therapeutic feedback, ultimately achieving more precise cancer diagnosis and treatment.

#### 2.1.1 Mt-targeted tumor imaging platforms

Mt are core organelles responsible for cellular energy metabolism and biosynthesis, maintaining intracellular homeostasis through the tricarboxylic acid cycle (TCA), lipid metabolism, and nucleic acid synthesis 26, 27. To meet the demands of continuous tumor cell proliferation, Mt undergo abnormalities such as metabolic reprogramming, redox imbalance, and functional deterioration, which are specifically manifested as the accumulation of metabolic intermediates, mitochondrial DNA (mtDNA) mutations, and altered respiratory activity 28. These pathological changes not only promote tumor initiation and progression but also provide a critical biological basis for the design of Mt-targeted diagnostic and therapeutic strategies.

A significant transmembrane potential difference exists across the inner mitochondrial membrane, which drives lipophilic cations to cross the cell membrane and accumulate within the mitochondrial matrix 29. Lipophilic cationic functional motifs, such as Triphenylphosphine (TPP), pyridinium, quaternary ammonium, indolium, and quinolinium derivatives, have been widely employed in the design of Mt-targeted tumor diagnostic imaging platforms 30. With a deeper understanding of tumor mitochondrial biology, these imaging platforms have progressively evolved from relying solely on ΔΨm-driven localization to incorporating more refined designs, including the integration of tumor-associated mitochondrial proteins, mtDNA, metabolic intermediates, and microenvironment-responsive mechanisms, to achieve more accurate tumor imaging diagnosis (**Table 2**).

TPP is an organophosphorus compound that is electrically neutral in its original form 43. Its mitochondrial targeting capability relies on the permanent positive charge and hydrophobic aromatic structure of its derivatives, which enables rapid membrane permeation followed by accumulation driven by the negative ΔΨm. In early studies, most mitochondrial fluorescent probes employed TPP as the targeting moiety; however, certain normal tissues with high metabolic activity also exhibit elevated membrane potentials, leading to the nonspecific accumulation of TPP-based probes 44. Based on this, the design strategies for TPP-based mitochondrial imaging platforms have shifted from simply enhancing mitochondrial enrichment efficiency toward improving stable mitochondrial retention and tumor selectivity.

Mito-NQ exemplifies both the advantages and limitations of conventional TPP-based designs. By optimizing the linkage between TPP and the fluorophore, the researchers introduced TPP into the non-conjugated region of the fluorophore scaffold, thereby minimizing its impact on the photophysical properties (**Figure 2A**). The probe exhibited a Pearson colocalization coefficient of 0.98 with MitoTracker and achieved a Tumor-to-Normal tissue ratio (T/N) fluorescence ratio of 3.0 in A549 tumor-bearing mice, demonstrating high mitochondrial localization at the cellular level and clear imaging of tumor boundaries at the tissue level 31. However, its tumor fluorescence contrast still mainly depends on differential probe accumulation of the probe, rather than the tumor- tumor-specific activation events.

Relying solely on membrane potential cannot achieve true tumor specificity. By introducing a second recognition mechanism that combines mitochondrial localization with tumor-associated molecular abnormalities, a dual-recognition system is established, further improving diagnostic accuracy. Yang *et al*. developed FE-T NPs, a Glutathione (GSH)-responsive NIR-II mitochondrial imaging platform (**Figure 2B**). The cleavage of surface disulfide bonds in response to elevated intracellular GSH levels endowed FE-T NPs with tumor-targeting capability. The incorporation of tetravalent TPP further enhanced the interaction between the probe and mitochondrial membranes. Combined with NIR-II imaging, this platform achieved a high signal-to-noise ratio in deep tissues and enabled synergistic PDT and PTT 32. However, the strategy still relies on membrane potential-driven accumulation. When mitochondrial dysfunction or depolarization occurs, targeting efficiency may decrease, and nonspecific membrane interactions caused by lipophilic cations persist.

Multivalent TPP can also be integrated with signal amplification strategies to improve the sensitivity of tumor diagnosis. Guo *et al*. developed the SRDN DNA nanoplatform, utilizing multivalent TPP to enhance mitochondrial binding. The platform employed mitochondrial 16S rRNA-triggered hybridization chain reaction (HCR) for signal amplification. A single target molecule induced the physical separation of hundreds to thousands of Cy5 fluorophores from BHQ2 quenching groups, resulting in significant fluorescence amplification. The platform achieved an ultralow in vitro detection limit (LOD) of 86.7 pM for miR-21, substantially lower than that of many conventional miRNA detection methods, representing an advancement in the sensitivity of mitochondrial biomarker detection. In MCF-7 tumor-bearing mice, the platform exhibited enhanced tumor accumulation and improved *in vivo* imaging signals 33.

Mitochondrial unfolded proteins response (mtUPR) mainly arises from protein folding defects or post-translational modifications of mitochondrial proteins 45. Under pathological conditions, these manifest as altered ΔΨm, mitochondrial hyperpolarization, and impaired protease activity and clearance capacity. The accumulation of mtUPR is directly associated with apoptosis resistance and metabolic adaptation, resulting in their abnormal accumulation in tumor cells 46. To further reduce ΔΨm dependence, researchers introduced endogenous mitochondrial molecular recognition mechanisms. Jun *et al*. developed the MAP probe by combining TPP-mediated mitochondrial targeting with mtUPR recognition capability (**Figure 2C**). TPP first facilitates probe entry into mitochondria, followed by covalent binding between the maleimide group and thiol groups within mtUPR, achieving stable probe retention. This dual-targeting strategy improved probe stability under membrane potential fluctuations, increased the fluorescence difference between tumors and normal tissues to 9.5-fold, significantly enhanced tumor selectivity, and enabled ovarian cancer tissue identification and surgical navigation 34.

Pyridinium salts represent another class of commonly used lipophilic cations. Driven by electrochemical principles, these lipophilic cationic small molecules can selectively cross phospholipid bilayers and accumulate extensively within the mitochondrial matrix, often reaching concentrations hundreds of times higher than those in the cytoplasm 47. In addition, their strong electron-accepting capability enables their integration into D-π-A fluorescent structures. Compared with TPP, which mainly serves as a localization moiety, pyridinium salts offer greater flexibility in optical regulation and the construction of responsive probes 48.

Guo *et al*. integrated a pyridinium structure with an azo reductase-responsive unit to develop a two-photon mitochondrial probe, TPNP. The pyridinium moiety enabled TPNP to achieve a Pearson colocalization coefficient of 0.9178 with Mt in tumor cells, demonstrating highly efficient mitochondrial targeting. Azo reductase-mediated cleavage of the azo bond released the fluorophore ASPI, producing a hypoxia-activated fluorescence turn-on response, thereby providing an effective strategy for the diagnosis of hypoxic tumors. Meanwhile, two-photon excitation further improved the imaging capability in deep tumor tissues 35.

To enhance mitochondrial targeting efficiency, Yu *et al*. proposed a dual-cation synergistic strategy by simultaneously introducing TPP and pyridinium groups into the TPAP2 probe (**Figure 2D**). The time required for tumor mitochondrial localization was reduced to 10 s, and the mitochondrial colocalization coefficient reached 0.93. Strong fluorescence signals were observed exclusively at the subcutaneous tumor site. Since the unbound probe generates almost no background fluorescence, this probe enables “wash-free” imaging, avoiding the fluorescence loss or cellular damage that may be caused by washing procedures 36.

The TAB-4-TP probe designed by Liu *et al*. does not directly utilize pyridinium salts as targeting motifs; instead, it employs the positively charged N-methyl-tetrahydropyridyl and piperazine groups within TAB-4-TP itself to electrostatically attract negatively charged mtDNA, thereby achieving mitochondrial targeting (**Figure 2E**). TAB-4-TP responds to the high concentration of hypochlorous acid (HClO) in tumor cells, generating a pyridinium-containing product with mtDNA-binding capability. Through this dual-targeting response strategy involving mtDNA and HClO, the platform can discriminate between tumor cells and normal cells 37.

In addition to enhancing the selectivity of mitochondrial localization, current research also focuses on exploiting the abnormal mitochondrial microenvironment for dynamic functional imaging. By integrating cationic targeting motifs with responsive fluorescent structures, probes can generate selective fluorescence signals in response to viscosity, ROS, gaseous signaling molecules, and metabolic abnormalities, thereby transforming mitochondrial imaging from simple localization into functional state monitoring. For example, Tian *et al*. developed Mito-BCy, employing a benzindolium quaternary ammonium cation (indolium iodide) as the mitochondrial targeting group (**Figure 2F**). By incorporating a molecular rotor structure, Mito-BCy enables viscosity-responsive imaging and can distinguish tumor tissues from normal tissues 38.

Compared with TPP and pyridinium salts, quinolinium cations possess larger conjugated systems and stronger electron-accepting capabilities, which facilitate long-wavelength fluorescence emission 49. Wang *et al*. developed DCMQ, employing a quinolinium cation for mitochondrial targeting and utilizing a D-π-A structure to respond to changes in tumor mitochondrial viscosity (**Figure 2G**), ultimately achieving high signal-to-noise ratio imaging in 4T1 tumor-bearing mice 21. However, the *in vivo* metabolism, biosafety, and integration of these emerging cationic motifs with active targeting strategies warrant further investigation.

The high metabolic activity of tumors also leads to significantly higher DNA content and concentration in the Nucleus compared with normal cells 50. Li *et al*. developed the BHD probe for dual-targeted mitochondrial-nuclear imaging (**Figure 2H**). This system initially relies on quaternary ammonium groups for mitochondrial entry, followed by nuclear translocation mediated by aromatic interactions with DNA, producing enhanced near-infrared fluorescence signals in tumor-bearing mice 39.

Subsequently, researchers introduced Hydrogen sulfide (H₂S)-responsive units into mitochondrial imaging platforms to improve response speed and *in vivo* imaging performance. The lipophilic cationic hemicyanine scaffold contained within HCy-SSPy drives its mitochondrial localization. By integrating the mitochondrial targeting structure with the rapid H₂S-responsive unit, HCy-SSPy enables real-time monitoring of dynamic H₂S fluctuations in pathological microenvironments (**Figure 2I**). The platform achieved a second-level response time (5 s) and *in vivo* imaging in tumor-bearing mice 40. Similarly, 152T introduces a biotin group onto the pyridine nitrogen atom to form a pyridinium salt-based mitochondrial targeting structure (**Figure 2J**). The steric hindrance provided by the biotin group improves the selectivity of H₂S recognition and enhances water solubility, enabling rapid retention and imaging of tumor tissues 41.

Adenosine Triphosphate (ATP) is a core molecule of cellular energy metabolism and serves as a critical energy source for maintaining mitochondrial oxidative phosphorylation and cellular activities. Tumor cells exhibit significantly higher rates of ATP synthesis and consumption than normal cells, particularly maintaining elevated ATP turnover within Mt 51. Therefore, ATP not only reflects cellular metabolic activity but can also serve as a metabolic biomarker to improve the tumor selectivity of Mt-targeted probes.

In 2023, the Lu team developed the near-infrared ratiometric ATP-responsive probe M838 (**Figure 2K**). The probe employed a rhodamine-cyanine hybrid scaffold containing indolium/xanthene structures with lipophilic cationic characteristics. A spirolactam ring-opening and ATP multivalent binding mechanism was introduced as the response switch, and a dual-channel ratiometric signal was constructed to improve the quantitative analysis of ATP, achieving a linear response range of 2.0-7.5 mM. The incorporation of the ATP-responsive unit enhanced the probe's specificity, anti-interference capability, dynamic monitoring ability, and tissue penetration depth. M838 enables dynamic monitoring of ATP fluctuations and achieves high T/N signal contrast in living tumor models 42.

Subsequently, the Yang team built the mitochondrial-targeted ATP response probe TPA-P4 (**Figure 2L**). The platform utilized a pyridinium structure for mitochondrial enrichment and employed a synergistic dual-recognition strategy combining "boronic acid-diol covalent binding" and "positive charge-triphosphate electrostatic attraction" to ensure a specific response to ATP within tumor cell mitochondria, with a detection limit reaching 23.8 nM. The probe exhibited significant fluorescence intensity differences between hepatocellular carcinoma cells and normal hepatocytes, enabling sensitive monitoring of subtle ATP variations under pathological stimuli, which is conducive to early tumor diagnosis 18.

The development of the mitochondrial-targeted tumor diagnostic imaging platform has undergone a gradual transformation from a single membrane potential-driven positioning to a multi-condition stimulation targeting strategy. Early strategies relied solely on the negative membrane potential of the inner mitochondrial membrane to drive the accumulation of lipophilic cations (such as TPP, pyridinium salts, quaternary ammonium salts, and quinolinium cations) to achieve rapid enrichment within Mt. TPP remains the most widely used mitochondrial targeting motif due to its structural stability, facile synthesis, and high mitochondrial uptake efficiency; however, such strategies inherently depend on ΔΨm differences and are prone to nonspecific mitochondrial localization. Subsequently, researchers enhanced probe retention through multivalent modification, dual-cation synergistic interactions, and endogenous mitochondrial molecular recognition. For example, FE-T NPs utilized multivalent TPP to enhance mitochondrial binding and incorporated GSH responsiveness for deep-tissue imaging. MAP achieved dual anchoring through TPP-mediated mitochondrial entry and covalent capture by mtUPR, increasing the tumor/normal tissue fluorescence difference to 9.5, significantly superior to conventional TPP platforms.

From the perspective of tumor diagnostic accuracy, simply improving mitochondrial enrichment efficiency may not translate into enhanced tumor selectivity. Based on the performance advantages of the above platforms, dual-diagnostic strategies integrating mitochondrial targeting and tumor-associated signal responsiveness exhibit greater diagnostic potential. For example, MAP reduces ΔΨm dependence through mtUPR recognition and achieves a higher T/N. TPNP and TAB-4-TP restrict fluorescence signals to tumor-associated mitochondrial environments through hypoxia or HClO/mtDNA dual-responsive mechanisms. The ATP-responsive platforms M838 and TPA-P4 exploit tumor metabolic abnormalities for functional imaging. As a result, using only TPP or other lipophilic cations is not the optimal design strategy; instead, priority should be given to dual-module designs that combine classical mitochondrial targeting motifs with tumor-specific molecular response units. This strategy not only ensures efficient mitochondrial entry of probes but also discriminates target cells by exploiting tumor-specific metabolic abnormalities, redox imbalance, or protein expression alterations, thereby improving T/N ratios, signal-to-noise ratios, and imaging reliability.

The development of Mt-targeted diagnostic platforms has primarily focused on balancing localization, responsiveness, and imaging performance. On the one hand, traditional lipophilic cations possess mature mitochondrial delivery advantages, and their further development may focus on reducing nonspecific membrane interactions, such as improving mitochondrial retention stability through multivalent interactions, reversible binding, or active targeting ligands. On the other hand, integrating mitochondrial targeting with tumor metabolic biomarkers, redox abnormalities, and Mt-specific molecules can further enhance diagnostic selectivity. Moreover, with increasing demands for deep-tissue diagnosis, NIR-II emission, ratiometric imaging, and dynamic functional monitoring are becoming important directions.

#### 2.1.2 Lysosome-targeted tumor imaging platforms

Lysosomes are single-membrane-bound organelles containing over 60 distinct acid hydrolases. Functioning as the cellular “digestive factories”, they are central to the degradation of macromolecules and senescent organelles, autophagy, antigen presentation, and energy metabolism 52. In malignant cells, the expression and secretion of multiple lysosomal hydrolases are markedly elevated 53, 54. These enzymes promote tumor angiogenesis, invasion, and metastasis 55 by activating specific signaling cascades, degrading the extracellular matrix (ECM), remodeling the cytoskeleton, and modulating the tumor microenvironment. Consequently, theranostic strategies targeting lysosomal enzymes are undergoing rapid development 56. Therapeutically, inducing Lysosomal membrane permeabilization (LMP) and engineering enzyme-responsive drug delivery systems have proven highly effective for Lysosome-targeted cancer interventions 57-59.

Currently, Lysosome-targeted tumor imaging strategies primarily rely on two approaches: one utilizes the acidic lumen to achieve organelle localization, whereas the other approach further incorporates tumor-associated enzymes, metabolites, or reactive species to enhance imaging selectivity 60. Among these strategies, morpholine is the most widely used Lysosome-targeting functional motif. However, because acidic Lysosomes are also present in normal cells, strategies solely relying on pH differences are insufficient to achieve tumor-specific imaging 61. Therefore, recent studies have gradually integrated Lysosome-targeting motifs with tumor-associated responsive units and adopted a “targeting-plus-activation” design to enhance signal differences between cancer cells and normal tissues. Representative lysosomal imaging platforms are compared in **Table 3**.

OELyso-Gal represents a “targeting-plus-activation” architecture. Morpholine mediates lysosomal accumulation, while the glycosidic bond, responsive to β-galactosidase (β-gal), controls the recovery of near-infrared fluorescence. Enzymatic cleavage releases the RNA-interacting fluorescent CyON structure (**Figure 3A**), thereby enhancing intracellular retention. In reported ovarian cancer experiments, this platform distinguished tissues with high and low β-gal expression and achieved a T/N of 74.6 for tumor boundary imaging, demonstrating excellent diagnostic performance and ultimately enabling tumor margin visualization 62. This high contrast highlights the contribution of enzyme-activated responses to diagnostic precision; however, its applicability remains limited in tumors with low or heterogeneous β-gal expression.

Multiconditional activation can further reduce background interference. H₂S is an important gas signal molecule in the body, which is abnormally elevated in a variety of tumor cells 67 and can be used as a new activation signal to participate in the design of imaging diagnostic platforms. In 2025, the Li's team developed a morphine-modified NIR-Lys-H₂S probe, which uses morphine to achieve lysosomal localization and uses the NBD structure of H₂S-responsive NBD structure to regulate the fluorescence output (**Figure 3B**). After entering H₂S-overexpressing tumor cells, H₂S induced cleavage of the NBD structure, restoring the fluorescent conjugated system and producing enhanced near-infrared fluorescence. The probe was capable of distinguishing U87 glioma cells from normal cells and enabled imaging in tumor-bearing mice 63.

In duNP-DA, the 2,3-dimethylmaleic anhydride group undergoes hydrolysis in the weakly acidic extracellular tumor environment at approximately pH 6.5 (**Figure 3C**), exposing positively charged amino groups to promote cellular uptake. Subsequently, lysosomal delivery and H₂S-dependent activation generated fluorescence signals in the 4T1 model and experimentally facilitated the identification of small metastatic lesions, with a reported T/N of 8.2 64. Compared with simple pH-based localization strategies, metabolite-responsive designs further exploit tumor metabolic differences to improve selectivity; however, H₂S levels are cell-type dependent, limiting their universal applicability.

The imbalance of redox homeostasis in tumor cells leads to elevated levels of reactive oxygen and nitrogen species 68. Combining lysosomal localization with oxidative stress responsiveness can further improve tumor recognition capability. Lyso-PE integrates morpholine-mediated localization with a Peroxynitrite anion (ONOO⁻)-responsive group. After cellular internalization, morpholine promotes lysosomal enrichment, followed by ONOO⁻-induced oxidative cleavage of the responsive group to restore fluorescence (**Figure 3D**). This probe was used to detect oxidative stress in 4T1 cells, visualize tumors in mice, and achieve tumor tissue imaging and fluorescence-guided surgery in tumor-bearing mice 65.

Traditional Lysosome-targeted fluorescent probes generally rely on weakly basic amine structures; however, some amine-based probes suffer from limitations, including pKa-dependent targeting efficiency, restricted structural modification space, and insufficient *in vivo* stability 69. Therefore, developing Lysosome-targeting motifs with novel chemical characteristics has become an important direction for improving lysosomal imaging performance. Unlike conventional weakly basic amine structures, SOH utilizes a positively charged sulfonium center and a lipophilic structure to accumulate in acidic organelles and achieves signal variation through pH-responsive fluorescence regulation (**Figure 3E–3F**). The probe rapidly localized to Lysosomes and exhibited high colocalization efficiency with a Pearson correlation coefficient of 0.87. However, this strategy primarily relies on acidic environmental differences, resulting in a T/N of only 2.5, indicating that simply replacing the targeting group cannot overcome the issue of tumor selectivity. It needs to be further combined with active targeting or tumor-responsive mechanisms to enhance its clinical application value 66.

Currently, Lysosome-targeted platforms still primarily use morpholine as the core targeting motif, and its mature “ion trapping” mechanism enables stable and efficient lysosomal enrichment 70. However, simple morpholine-based targeting is insufficient to meet the requirements of tumor-specific imaging. Therefore, current studies are gradually shifting toward a dual “targeting-plus-response” design strategy, in which tumor-associated biomarkers are integrated to activate fluorescence signals specifically within tumor cells. This approach significantly improves discrimination between cancer cells and normal cells and further expands applications in integrated PDTand intraoperative navigation. In the future, combining NIR-II fluorescence, multi-responsive cascade activation, and tumor-targeting ligands may further improve deep-tissue imaging capability while reducing nonspecific accumulation caused by acidic vesicles, thereby promoting the clinical translation of Lysosome-targeted platforms toward precision imaging and therapy.

#### 2.1.3 ER-targeted tumor imaging platforms

The ER is a membrane-bound organelle in eukaryotic cells that plays pivotal roles in protein synthesis, folding, transport, lipid synthesis 71, and intracellular Ca²⁺ homeostasis. Under microenvironmental stress—such as hypoxia, oncogene activation, or tumor suppressor gene loss—cells trigger the ERs response 72. This response initially activates three primary transmembrane sensors: IRE1, PERK, and ATF6 73. If stress remains unresolved, these sensors subsequently activate downstream pro-apoptotic mediators—such as caspase-12, JNK, and CHOP—to initiate programmed cell death, thereby preventing prolonged ER dysfunction. Conversely, in cancer cells, chronic hypoxia and nutrient deprivation often hijack the unfolded protein response (UPR) to activate protective ERS pathways (e.g., IRE1/XBP-1 and PERK), thereby promoting tumor cell survival, proliferation 74, and immune evasion 75. However, the excessive activation of UPR can break through the tolerance range of this protection mechanism, thus inducing tumor cell apoptosis and exerting a strong anti-tumor effect 76. Beyond ERs regulation, the aberrant expression of various ER-resident proteins also drives tumor angiogenesis and correlates with poor clinical prognosis 77.

Unlike Mt and Lysosomes, which rely on membrane potential and acidic environments for targeting, respectively, the ER lacks distinct physicochemical localization characteristics. Consequently, ER-targeted imaging relies primarily on specific functional motifs for the molecular recognition of ER membrane proteins, or utilizes the lipid environment of ER membranes and tumor-associated signals to achieve selective enrichment 78. Currently, the major strategies include (i) active targeting based on Sulfonylurea Receptor (SUR) recognition, (ii) passive enrichment based on membrane lipid interactions, and (iii) activatable imaging strategies that incorporate tumor-specific responsive units. Representative ER imaging platforms are compared in **Table 4**.

p-Toluenesulfonamide is one of the most rapidly advancing ER-targeting functional motifs and has been widely adopted to construct ER-targeted fluorescence imaging platforms, owing to its ability to bind to SURs on the ER 82. This motif specifically recognizes SUR proteins on the ER membrane, facilitating the selective accumulation of probes within the ER. Through the interaction between the p-toluenesulfonamide unit and SUR, and the optimization of the fluorescent scaffold, this design enables both effective ER localization and near-infrared emission (**Figure 4A**). The resulting probe exhibited a Pearson correlation coefficient of 0.964 with ER-Tracker and achieved a reported cancer-to-normal cell fluorescence ratio of approximately 2, enabling ER fluorescence imaging in living cells and tumor-bearing mouse models, as well as preliminary discrimination between tumor and normal cells at the cellular level 79.

EICy-P further incorporated a CYP2J2-responsive unit into the same targeting framework. In CYP2J2-overexpressing tumor cells, enzyme-mediated transformation concurrently restored fluorescence and photodynamic activity (**Figure 4B**), thereby integrating ER localization with tumor-associated enzyme activation and enabling the discrimination of hepatocellular carcinoma cells from normal hepatocytes 80. Nevertheless, SUR-mediated localization depends on receptor expression levels, which may vary among cell types and compromise imaging performance. Therefore, ER-targeting strategies relying solely on protein recognition require additional tumor-selective mechanisms to ensure stability and reliability *in vivo*.

Te-BOD, by contrast, employs a highly lipophilic structure for membrane enrichment and utilizes a GSH-responsive tellurium-containing moiety for fluorescence activation, thus reducing dependence on specific ER receptors. The introduction of a sterically hindered phenyl-tellurium (PTE) group elevated the GSH response threshold of Te-BOD to 100 μM (**Figure 4C**), thereby restricting fluorescence activation to the high GSH concentrations characteristic of tumor cells. This GSH-dependent localization strategy achieved a high T/N of approximately 8–9 and successfully distinguished lung cancer tissues from adjacent normal tissues in *ex vivo* clinical specimens (T/N ≈ 3) 81. Although membrane-based ER localization reduces reliance on specific protein expression, its physicochemical origin means that additional tumor-responsive mechanisms are still needed to further suppress background signals from normal tissues.

Owing to the absence of clear endogenous localization signals, ER-targeted imaging currently relies on two principal strategies: molecular recognition and membrane environment modulation. Among them, p-toluenesulfonamide achieves high ER localization efficiency through SUR binding and remains one of the most established functional motifs. Lipophilic structures and bile acid-based strategies, in contrast, exploit differences in ER membrane composition to achieve nonspecific enrichment. In recent years, ER-targeted platforms have progressively transitioned from simple organelle localization toward a “targeting-plus-tumor-response activation” mode by integrating tumor-associated signals such as CYP2J2 and GSH, thereby enhancing the imaging contrast between cancer cells and normal tissues. Future efforts that integrate active tumor-targeting ligands, NIR-II imaging, and multi-responsive mechanisms hold promise for overcoming the current limitations in deep-tissue imaging and *in vivo* applications.

#### 2.1.4 Nuclear-targeted tumor imaging platforms

The Nucleus serves as the genetic command center of eukaryotic cells. The nuclear pore complex (NPC) mediates nucleocytoplasmic transport of RNA and proteins, while the compartmentalized chromatin harbors genetic information, precisely orchestrating essential life processes, including cell growth, division, differentiation, and apoptosis 83. Genomic instability in the Nucleus of tumor cells frequently manifests as defects in DNA damage repair mechanisms, which impede timely and accurate genomic restoration 84. This deficiency catalyzes the accumulation of genetic mutations, ultimately driving malignant transformation. In addition, the abrogation of cell cycle checkpoints and the dysregulation of epigenetic modifications exacerbate this genomic instability. Structural aberrations in the nuclear envelope and nuclear pore complexes can induce chromosome fragmentation, significantly complicating DNA repair processes 85.

To achieve nuclear targeting, the diagnostic platform must sequentially traverse the plasma membrane and the nuclear pore complex. Consequently, nuclear targeting primarily relies on nuclear localization sequences (NLS), recognition of nuclear proteins, electrostatic or minor groove binding to DNA, as well as tumor-associated antibodies 86. These approaches exhibit substantial differences in their mechanisms and supporting evidence. Among them, NLS is the most widely used functional motif for nuclear delivery; its classical sequence is derived from the simian virus 40 (SV40) large T antigen. NLS can be recognized by the importin α/β transport system and transported into the Nucleus through the NPC 87. Thus, modifying NLS onto the surface of fluorescent molecules or nanocarriers can improve the nuclear delivery efficiency of probes 88, 89. NLS improves nuclear transport but lacks tumor specificity. DNA-binding groups enable stable nuclear retention but may also interact with normal genomes. Protein ligands and antibodies may enhance disease selectivity but are limited by target heterogeneity and delivery efficiency. Researchers have developed imaging platforms that combine nuclear localization capability with tumor recognition by targeting tumor-associated nuclear proteins, DNA structural features, and abnormally expressed nuclear molecules. Representative nuclear imaging platforms are compared in **Table 5**.

NLS-mediated nuclear localization strategies utilize the natural nuclear transport machinery to improve the delivery efficiency of fluorescent molecules and drug carriers. The CDs-NLS-DOX nuclear-targeted delivery system achieves nuclear localization through the classical NLS sequence PKKKRKVG and loads Doxorubicin (DOX) via acid-sensitive hydrazone bonds (**Figure 5A**). NLS guides the platform into the Nucleus through the NPC, enabling intracellular nuclear enrichment of both carbon dots and DOX 93. Although NLS enhances nuclear delivery efficiency, it cannot distinguish tumor cells from normal cells. For imaging platforms aiming to improve tumor selectivity, NLS alone often requires combination with tumor-specific recognition units to reduce nonspecific accumulation in normal tissues.

Compared with NLS strategies that depend on universal nuclear transport mechanisms, the recognition of overexpressed nuclear proteins can provide stronger tumor discrimination capability. DSPC-PBP employs a BODIPY fluorophore and a pyrimidine ligand with affinity for Thymidylate Synthase (TYMS), a DNA synthesis enzyme that is elevated in rapidly proliferating tumors (**Figure 5B–5C**). The DSPC-PBP probe achieved a nuclear colocalization coefficient of 0.9 in HCT116 colon cancer cells, with an average fluorescence intensity approximately 2.1-fold higher than that in normal control cells, significantly exceeding the signals observed in other normal tissues 90. This strategy ties nuclear retention to proliferation-associated targets; however, differences in TYMS expression may limit its applicability across different tumor types.

Direct DNA binding represents a targeted imaging strategy that does not rely on active nuclear transport. The near-infrared probe 1HA4CD contains pyridinium and quaternary ammonium groups, which enhance its binding affinity to the DNA minor groove (**Figure 5D**). Upon entering tumor cells, the probe binds to nuclear DNA and remains in the Nucleus, while achieving high signal-to-noise ratio imaging via near-infrared fluorescence. The probe generated stable nuclear fluorescence, with a 10.6-fold signal difference between MDA-MB-231 cancer cells and HK2 normal cells 91. This DNA-binding strategy reduces dependence on complex nuclear transport processes while providing stable nuclear localization signals. However, since DNA is ubiquitously present in normal cell nuclei, the cationic structure may induce nonspecific binding; therefore, further optimization via tumor-selective response mechanisms remains necessary.

Antibody-mediated nuclear targeting strategies utilize the abnormal expression of tumor-associated nuclear proteins to achieve highly specific recognition. Survivin (BIRC5, Baculoviral IAP Repeat Containing 5) is a typical tumor-associated protein that is markedly elevated in the nuclei of various malignant tumor cells, including colorectal cancer cells. Antibodies against Survivin can serve as nuclear-targeting recognition units to improve probe selectivity toward tumor tissues. In the reported PVP@PNCs@Ab study, antibody-based Survivin recognition enabled *ex vivo* colorectal tissue analysis (**Figure 5E**), with the average fluorescence intensity of cancer tissue sections approximately 8.7-fold higher than that of adjacent normal tissues, providing a high signal-to-noise ratio 92. However, antibody-based nuclear-targeted imaging platforms generally have large molecular sizes, resulting in limited penetration across cellular and nuclear membranes. In addition, their high preparation costs restrict further *in vivo* applications. Nuclear-targeted imaging strategies have primarily developed around two directions: improving nuclear delivery efficiency and enhancing tumor selectivity. NLS utilizes natural cellular transport mechanisms to achieve efficient nuclear delivery but lacks tumor-specific recognition capability. Abnormally expressed nuclear molecules such as TYMS and Survivin provide new targets for selective imaging. DNA-binding strategies rely on stable nuclear retention to achieve persistent imaging and are suitable for theranostic platforms. Currently, nuclear-targeted imaging still faces two major limitations. On the one hand, strategies solely relying on nuclear localization mechanisms may lead to nonspecific accumulation in normal tissues. On the other hand, barriers from the nuclear envelope and plasma membrane limit the delivery efficiency of some macromolecular probes. Future studies should further integrate tumor-specific molecular recognition, responsive design, and highly penetrative imaging technologies to improve the selectivity and *in vivo* performance of nuclear-targeted platforms.

#### 2.1.5 GA-targeted tumor imaging platforms

The GA is an essential organelle involved in protein processing, glycosylation modification, lipid transport, and vesicle sorting. During tumor development, its structure and function are frequently remodeled, leading to abnormal glycosylation, enhanced vesicle trafficking, and membrane dynamic alterations 94, 95. These alterations are closely associated with tumor proliferation, invasion, and immune escape. Therefore, developing selective imaging strategies targeting GA abnormalities may facilitate tumor cell identification and monitoring of disease-associated functional states.

Unlike Mt and Lysosomes, which rely on membrane potential and acidic environments for targeting, respectively, GA lacks distinct physicochemical characteristics for organelle localization. Current probes primarily exploit interactions between GA-associated proteins, enzymes, and membrane environments. Common targeting motifs include indomethacin and celecoxib, which can bind cyclooxygenase-2 (COX-2), benzenesulfonamide derivatives, and peptide substrates of GA-associated enzymes such as furin 96. These targeting units can be integrated with fluorescence responses associated with molecular motion, viscosity, polarity, reactive species, or enzymatic cleavage. Representative GA imaging platforms are compared in **Table 6**.

Cyclooxygenase-2 (COX-2) is an inflammation-associated inducible enzyme that is aberrantly overexpressed in various tumor cells and participates in prostaglandin E₂ (PGE₂) synthesis, tumor proliferation, and immune escape 103. Some COX-2-selective inhibitors can bind COX-2 and thus serve as GA-associated tumor recognition units to guide selective probe localization. Among them, Indomethacin (IMC) and Celecoxib (CXB) are currently the two most extensively studied functional motifs. As a typical Nonsteroidal Antiinflammatory Drugs (NSAID), IMC has been widely used to construct GA-targeted imaging platforms due to its selective binding capability to COX-2 104. In 2024, the Qi's group reported an AIE-based GA-targeted theranostic probe, TTPI. TTPI integrates indomethacin with a triphenylamine–pyridinium AIE scaffold (**Figure 6A**). In COX-2-overexpressing tumor cells, binding restricts intramolecular motion and enhances fluorescence, while the same photophysical design also supports ROS generation 97. Within an imaging framework, TTPI demonstrates that GA-associated recognition ligands can simultaneously promote localization and enhance emission.

In addition to IMC, the sulfonamide structure and hydrophobic aromatic region of CXB can also participate in COX-2 binding. NP-C6-CXB connects celecoxib with a naphthalimide fluorophore through a six-carbon linker (**Figure 6B**). COX-2 binding inhibits photoinduced electron transfer and restores fluorescence, achieving a reported cancer/normal cell ratio of 7.9. The imaging depth of the probe in tumor tissue reached approximately 500 μM 98.

In addition to COX-2 inhibitors, studies have shown that the benzenesulfonamide structure within SC-558 itself can serve as a targeting functional motif for the GA. The introduction of this benzenesulfonamide structure simplifies the design of Golgi-targeted probes, offering greater structural flexibility 105. The differences in Golgi viscosity and polarity in cancer cells, combined with the benzenesulfonamide group, can further enhance tumor diagnostic capability.

GOL-V utilizes a BODIPY molecular rotor and a TICT mechanism to reflect increased GA viscosity (**Figure 6C**). The reported fluorescence ratio between cancer and normal cells was 7, and the tumor/normal tissue fluorescence ratio was approximately 3. The probe was evaluated in tumor-bearing mice and *ex vivo* lung cancer and thyroid cancer surgical specimens 99. GCSP similarly utilizes Intramolecular Charge Transfer (ICT) to report GA polarity changes. By incorporating a coumarin 343 polarity-responsive structure, GCSP enables GA polarity detection through the ICT process (**Figure 6D**). It can be applied to analyze GA functional states in living cells, tissues, and surgical samples from cancer patients 100. These platforms demonstrate the potential of organelle functions for tumor imaging; however, viscosity and polarity may also be affected by factors beyond malignant transformation.

Cancer cells remain under chronic oxidative stress, resulting in persistently elevated HClO levels compared with normal tissues. Combining GA localization with HClO monitoring can improve tumor selectivity 106. In 2024, the groups of Wang and Liang reported a GA-targeted HClO-responsive near-infrared probe, GA-BOD-S. GA-BOD-S uses a benzenesulfonamide group for GA targeting and activates near-infrared fluorescence through an HClO-responsive switch (**Figure 6E**). This strategy increased the signal difference between tumor and normal tissues and was applied for fluorescence-guided imaging of peritoneal metastatic tumors. However, its validation in clinical samples and deep-tissue imaging capabilities require further evaluation 101.

Enzyme-responsive peptides provide another design strategy. Furin is a GA-localized protease that is aberrantly expressed in various tumor cells and can specifically recognize and cleave the Arg-X-Lys/Arg-Arg (RX(K/R) R) sequence. RF-Cou contains a Furin recognition sequence (**Figure 6F**). The probe connects a coumarin fluorophore with the Furin-recognition peptide Ac-Arg-Val-Arg-Arg-Lys-Phe-Phe-OH. After enzymatic cleavage, amphiphilic fragments are generated, promoting excimer formation, fluorescence red-shift, and lifetime extension. This enables GA-associated enzyme-responsive imaging and allows discrimination between Furin-high-expressing 4T1 cells and normal cells 102. However, the response time of the platform is approximately 6 h, and comprehensive *in vivo* efficacy data remain lacking. Furthermore, the GA localization mechanism has not been thoroughly validated. As such, enzyme responsiveness alone is insufficient to establish the organelle specificity of the platform.

In addition to the five classical organelles mentioned above, lipid droplets (LDs) have attracted increasing attention due to their critical roles in tumor metabolic reprogramming, inflammation, and ferroptosis. Recent studies have demonstrated that precise tumor diagnosis can be achieved through multiparametric responses to the lipid droplet microenvironment. Sun *et al*. developed an AIE-active dual-channel near-infrared probe, TPA-DCN-TPE 107, which achieves efficient lipid droplet targeting through its high lipophilicity (log P = 7.77). TPA-DCN-TPE possesses independently orthogonal dual-signal response channels. Viscosity-induced restriction of intramolecular motion (RIM) activates the red fluorescence channel, resulting in a 1532-fold enhancement of fluorescence intensity, whereas the specific oxidation reaction with HClO activates the green fluorescence channel, producing a 363-fold fluorescence enhancement. This ratiometric Red/Green Fluorescence Signal (R/G ratio) enables the platform to distinguish early-stage hepatocellular carcinoma (HCC, R/G > 1.5) from acute liver injury (ALI, R/G < 0.8), allowing accurate discrimination between these two pathologically similar conditions both *in vivo* and at the tissue-section level.

In a related study from the same research series, Zhan *et al*. further reported the BCz-DCN-TPA probe 108. This platform not only retained lipid droplet-targeting capability and independent dual-channel responses to viscosity (391-fold enhancement) and HClO (422-fold enhancement) but also creatively integrated Photoacoustic Imaging (PAI) functionality. In a subcutaneous hepatocellular carcinoma model, the probe provided near-infrared fluorescence feedback on pathological alterations in the lipid droplet microenvironment after intravenous administration. Meanwhile, owing to its excellent near-infrared absorption properties, the probe clearly delineated tumor boundaries and morphology in deep tissues, enabling the integration of subcellular microenvironmental signals from fluorescence imaging with macroscopic tissue-level structural information from photoacoustic imaging. The design of these probes indicates that multiparametric and multimodal signal acquisition in organelle-targeted diagnostics is becoming an important trend for improving the precision of early cancer diagnosis.

Overall, relying solely on the physicochemical properties of organelles for subcellular localization, such as lipophilic cation-based mitochondrial targeting and weakly basic group-based lysosomal targeting, can achieve high localization efficiency but may also result in accumulation in normal organelles. ER, nuclear, and GA probes can employ more specific molecular recognition strategies, but their universality is limited by receptor or enzyme heterogeneity, while nuclear delivery introduces additional safety and transport barriers. Therefore, more convincing tumor-selective imaging designs generally combine established organelle-targeting mechanisms with independent tumor-associated recognition or activation events.

Regarding tumor diagnostic accuracy, the key factor determining imaging selectivity is whether tumor tissues can be effectively distinguished from normal tissue backgrounds. Platforms with dual or multiple recognition mechanisms generally exhibit superior diagnostic performance. In mitochondrial imaging, although the traditional TPP-based probe Mito-NQ possesses good mitochondrial localization ability, its T/N value is only 3.0; in contrast, the MAP platform, which integrates mtUPR recognition, achieves a T/N of 9.5 through secondary anchoring, significantly enhancing tumor selectivity. In the metabolite-responsive platform, TPA-P4 further restricts signals to highly metabolically active tumor cells via an ATP dual-recognition mechanism, achieving a detection limit of 23.8 nM. Studies targeting other organelles also demonstrate that combining organelle localization with tumor-associated abnormal events (such as elevated ROS, enzyme overexpression, protein homeostasis imbalance, and pH changes) can effectively reduce nonspecific background signals. Always-on fluorescent probes primarily determine localization, activatable probes reduce background signals, while ratiometric and reversible probes are more appropriate for quantitative and dynamic monitoring. The design of diagnostic platforms should not only consider colocalization coefficients or overall signal intensity but also evaluate tumor/normal tissue fluorescence contrast, activation dependence, reversibility, tissue depth, pharmacokinetics, safety, and validation scale.

Therefore, from the perspective of designing next-generation organelle-targeted diagnostic platforms, simply optimizing a single classical functional motif is not the optimal strategy. Instead, a multifunctional design integrating tumor-prioritized recognition, precise organelle localization, and specific signal activation should be adopted. Tumor-associated targeting units improve overall tissue selectivity, organelle-targeting motifs achieve subcellular spatial localization, and response modules ensure that signals are generated only within tumor pathological environments.

## 3. Organelle-targeted imaging for cancer theranostics

The integration of molecular imaging with therapeutic intervention has fundamentally transformed fluorescence-guided cancer treatment, elevating it from a diagnostic tool to a functional strategy that actively regulates therapeutic precision. In the preceding sections, we primarily discussed the structural basis of organelle-targeted tumor imaging platforms and the role of organelle localization in enhancing tumor imaging contrast and identifying pathological alterations. On this basis, this chapter focuses on how imaging information derived from organelle-targeted platforms can be leveraged to guide cancer therapy. These probes can not only visualize the distribution of therapeutic agents within tumors and subcellular structures but also determine the activation status of the theranostic platform, the optimal irradiation timing, and post-treatment organelle functional changes. Unlike the research that relies only on colocalization results to prove tumor targeting, the imaging-guided diagnosis and treatment integration emphasizes the relationship between imaging signals and treatment timing, intracellular transport dynamics, organelle damage extent, and treatment outcomes 109.

This chapter analyzes the role of organelle-targeted imaging in treatment timing selection, probe activation, subcellular transport monitoring, organelle damage evaluation, and immune response assessment, structured according to the imaging information required before, during, and after treatment.

### 3.1 Fluorescence imaging-guided cancer treatment strategies

Fluorescence imaging guides multiple stages of cancer treatment. In the early stage, fluorescence signals can monitor the accumulation of drugs within the tumor, and researchers determine the appropriate irradiation time window based on the degree of accumulation 110. During treatment, activatable or multichannel fluorescence signals provide real-time dynamic information regarding probe delivery, therapeutic activation, and intracellular distribution. After treatment, abnormal organelle function typically indicates therapeutic responses earlier than changes in tumor volume. This chapter primarily analyzes the key role of fluorescence imaging at different stages of cancer therapy.

#### 3.1.1 Determination of treatment timing

The efficacy of PDT and PTT depends not only on the accurate delivery of the probe to the tumor site but also on the successful cellular uptake of the photosensitizer and its accumulation in specific organelles prior to irradiation. Different delivery systems exhibit distinct circulation kinetics, tumor retention characteristics, and intracellular activation dynamics; fixed-time irradiation may result in insufficient accumulation or premature degradation of therapeutic agents.

Chen *et al*. constructed LipoHPM. Upon entering tumor cells, the platform dissociated in the acidic lysosomal environment, simultaneously restoring its fluorescence and ROS generation capabilities. Based on the *in vivo* fluorescence changes, the study determined that 12 h post-administration was the optimal irradiation time, thereby synchronizing the initiation of treatment with tumor accumulation and probe activation 111. In contrast, CYPT NPs utilized near-infrared fluorescence to track *in vivo* biodistribution (**Figure 7A**) and selected 24 h post-injection as the PDT/PTT treatment time, during which GSH-responsive release, ROS generation, and the photothermal effect collectively enhanced therapeutic efficacy 112. In the M@P system, the NIR signal of MTCN-3 was also employed to monitor tumor accumulation and assist in selecting irradiation timing (**Figure 7B**), ensuring that lysosomal damage occurred only after effective delivery of the theranostic platform 113.

These studies demonstrate that determining the therapeutic time window cannot rely solely on empirical judgment or isolated pharmacokinetic measurements. For theranostic platforms with responsive release or activation mechanisms, it is essential to distinguish the overall probe/drug accumulation signals from true therapeutic activation signals. At the same time, normal tissue background, signal duration, release kinetics, and final treatment outcomes must be considered. Only when imaging results can directly inform the optimal irradiation timing, adjust treatment plans, or exclude unsuitable treatment windows can fluorescence imaging truly fulfill its guiding role in therapy.

#### 3.1.2 Monitoring of probe activation and intracellular trafficking

After reaching the tumor site, the therapeutic agent must undergo a series of complex intracellular processes, including endocytosis, retention within organelles, structural transformation, and inter-organelle migration 114. As a result, a single colocalization analysis performed before treatment is insufficient to fully confirm that the therapeutic components have been activated, nor can it capture dynamic changes in their functional localization over time. In contrast, activatable or ratiometric fluorescent probes can correlate specific microenvironmental responses with fluorescence changes, thereby distinguishing tumor accumulation, intracellular release, and therapeutic activation as separate events. This type of dynamic fluorescence signal can reflect the specific time at which the therapeutic material transitions into an active state, rather than merely demonstrating its fluorescence imaging capability.

For theranostic systems involving multi-organelle or sequential targeting, changes in fluorescence spectra and spatial redistribution can further record dynamic transitions of therapeutic materials during treatment. THTTPy initially localizes primarily in Lysosomes and lipid droplets, emitting green fluorescence; upon light activation, it is converted into TTPy, which emits red fluorescence and redistributes to Mt. This green-to-red fluorescence conversion not only reflects the transformation of the molecular structure but also reveals the spatiotemporal relocation of the functional site, thereby enabling continuous tracking of early lysosomal damage and subsequent mitochondrial effects 115. Compared with single-time-point colocalization analysis, this dynamic imaging strategy better reflects the temporal progression of therapeutic materials. However, when interpreting spectral changes, the potential effects of molecular aggregation states and probe metabolism should still be carefully considered.

The multimodal imaging strategy can further compensate for the limitations of single fluorescence signals in terms of spatial resolution and functional information. PTQ-TPA3 employs fluorescence imaging to evaluate intracellular localization before treatment. Photoacoustic imaging provides tissue-scale distribution information (**Figure 7C**), and photothermal imaging provides real-time temperature feedback during irradiation. 116. Therefore, transport monitoring during therapy should assess not only whether the therapeutic materials exhibit organelle colocalization but also when they reach the target site, whether activation has been completed, and whether functional localization changes over time, thereby enabling imaging information to effectively guide the therapeutic process.

#### 3.1.3 Assessment of organelle damage and treatment response

Changes in tumor volume usually take a relatively long time to become apparent, whereas organelle dysfunction can often reflect therapeutic responses at a much earlier stage. Organelle-associated alterations—such as reduced ΔΨm, redox imbalance, LMP, ERs, and DNA damage—can serve as important early indicators for evaluating tumor treatment efficacy 117. Unlike imaging signals that merely reflect the distribution of materials, these indicators directly represent changes in the functional state of organelles, making them more suitable for determining whether therapeutic agents have successfully acted on the intended targets and achieved effective intervention levels.

Compound 17 maintained NIR fluorescence signals for over 120 h and correlated fluorescence changes with ROS accumulation, DNA damage, and cell fate transitions through long-term imaging tracking (**Figure 7D**), providing an example of continuous therapeutic response monitoring 118. For other organelle-targeted theranostic platforms, imaging is generally used first to confirm material delivery, subcellular localization, or irradiation processes, followed by evaluation of early therapeutic effects using membrane potential probes, LMP assays, ER stress-related protein analysis, and DNA damage markers. This multiparameter evaluation strategy can demonstrate whether treatments affect target organelle functions, but it remains difficult to directly quantify organelle damage levels through fluorescence signals from a single therapeutic probe.

It should be emphasized that most current organelle functional changes are still assessed through independent biological assays—such as membrane potential fluorescent dyes, protein expression analysis, DNA damage marker detection, and post-treatment histological evaluation—rather than via real-time reporting by the therapeutic probes themselves. Therefore, a more accurate description at the current stage is that imaging technologies are used to determine therapeutic material delivery and treatment timing, while independent functional indicators are applied to validate organelle damage and therapeutic outcomes. Future studies should further establish quantitative relationships between fluorescence intensity, ratiometric changes, or fluorescence lifetime parameters and organelle functional indicators—including membrane potential alterations, lysosomal membrane integrity, ER stress levels, and DNA damage extent—thereby advancing organelle imaging from localization and observation tools toward functional therapeutic feedback platforms.

### 3.2 Organelle-targeted phototherapy

Fluorescence-guided PDT is the most common modality in organelle-targeted theranostic systems. Currently, fluorescence imaging-guided Photodynamic Therapy (FIG-PDT) has also emerged as one of the most representative integrated therapeutic modalities. FIG-PDT exploits the dual functional role of photosensitizers (PSs), which simultaneously generate fluorescence signals for real-time visualization and ROS for tumor ablation upon light irradiation 119. This unique “see-and-treat” capability enables selective, minimally invasive, and spatially controllable cancer therapy while largely avoiding the development of drug resistance. More importantly, the therapeutic value of fluorescence imaging has extended beyond tumor localization. Based on the information encoded by fluorescence signals, imaging can determine optimal irradiation windows, continuously monitor therapeutic progression, validate key treatment events, and guide precise subcellular targeting, thereby collectively improving therapeutic accuracy and mechanistic understanding in comprehensive cancer therapy. The differences among various systems lie not only in their targeting sites but also in whether imaging actively contributes to in determining irradiation timing, therapeutic modality, and efficacy evaluation. The following sections discuss single-organelle targeting, sequential and multi-organelle targeting, and imaging-guided combination therapy, respectively. **Table 7** summarizes the imaging readouts, decision-making roles, functional evidence, and classification of evidence of representative therapeutic systems (**Figure 8**).

#### 3.2.1 Single-organelle-targeted phototherapy

The effects of single-organelle-targeted phototherapy primarily depend on the cellular functional processes disrupted by photodamage. Mt-targeted therapies generally rely on localized ROS accumulation, which subsequently disrupts ΔΨm, electron transport chain function, and energy metabolic homeostasis. For example, Mito-IsTp utilizes fluorescence signals to confirm mitochondrial delivery before treatment, generates both Type I and Type II ROS upon irradiation (**Figure 9A**), and further induces mitochondrial structural disruption and membrane potential reduction 120.

Chen *et al*. recently developed an activatable phototheranostic platform, NO₂/BDP-BT, through an integrated molecular design strategy. The platform combines a pyridinium-functionalized BODIPY core for mitochondrial targeting, a biotin unit for tumor-selective accumulation, and an NTR-responsive nitro group for hypoxia-associated activation 121. Upon enzymatic activation, NO₂/BDP-BT simultaneously enables fluorescence enhancement for hypoxia monitoring and activates Type I/II ROS generation (**Figure 9B**), thereby inducing mitochondrial dysfunction and apoptosis under hypoxic tumor conditions. Importantly, this platform further demonstrated enhanced antitumor immune responses, establishing a mechanistic connection between tumor-specific activation, mitochondrial damage, and therapeutic outcomes. Such activatable mitochondrial phototheranostic systems represent an important evolution from passive organelle targeting toward biologically regulated precision therapy.

Lysosome-targeted tumor therapy primarily amplifies cellular damage by inducing LMP. LMP can activate multiple cell death pathways by releasing hydrolytic enzymes, impairing autophagic function, and inducing secondary mitochondrial damage.

ER-targeted therapy primarily exerts its effects by disrupting protein homeostasis and inducing persistent ER stress. Ru1 confirmed its ER enrichment through colocalization imaging and further induced ER stress after irradiation, activating Cyclic GMP-AMP Synthase (cGAS-STING) and GSDMD-related pathways 123. In addition, compound 3 integrates two-photon imaging with a Type I PDT strategy (**Figure 9C**), maintaining the ability to induce ER stress under hypoxic conditions and subsequently causing secondary mitochondrial dysfunction 124. Nucleus-targeted therapy can directly act on genetic materials and induce oxidative DNA damage, but it demands higher delivery selectivity and biosafety. For example, UCNP@Glu-DMMA employs upconversion fluorescence signals to monitor the delivery process while enhancing nuclear accumulation through tumor-responsive charge conversion 125. For Nucleus-targeted therapeutic systems, imaging information should not only demonstrate nuclear localization but also establish correlations with intranuclear drug release efficiency and the extent of DNA damage.

Overall, Mt and the Nucleus, as key organelles directly regulating energy metabolism and genetic information, generally exhibit damage effects that are easier to observe and validate. In contrast, lysosomal and ER damage more often relies on signal amplification after local structural disruption and downstream stress responses. It should be noted that fluorescence signals in most current organelle-targeted therapeutic systems primarily reflect material distribution and localization, rather than directly quantifying changes in membrane potential, LMP levels, ER stress intensity, or DNA damage. Therefore, when evaluating organelle-targeted theranostic platforms, it is necessary to clearly distinguish among three different levels: “imaging-based validation of targeting localization”, “imaging-assisted therapeutic processes”, and “imaging-guided therapeutic decision-making”, thereby avoiding overestimating the actual significance of theranostic integration solely based on organelle colocalization results.

#### 3.2.2 Sequential and multi-organelle-targeted phototherapy

Single-organelle damage is not an isolated event, as the resulting metabolic disturbances, calcium signaling alterations, and death-related signals can further affect the functions of other organelles. Therefore, multi-organelle-targeted therapeutic strategies can generally be classified into two categories: synchronous localization and sequential migration. ADB can simultaneously localize to Mt and Lysosomes (**Figure 9D**), enabling synergistic ROS-mediated damage in two sensitive subcellular regions. Its fluorescence colocalization results can confirm dual-organelle distribution; however, direct feedback regarding the sequence of damage occurrence and organelle functional responses remains lacking 22. Such synchronous targeting strategies help expand the range of ROS action and enhance cellular damage but may also increase the risk of nonspecific phototoxicity and make it difficult to determine the specific contribution of damage to different organelles in the final therapeutic outcome.

In contrast, sequential targeting strategies utilize light-activation-induced molecular structural changes or organelle damage-associated processes to regulate probe redistribution, allowing imaging signals to dynamically change along with the therapeutic process. For example, THTTPy can migrate from initially targeted lysosomal and lipid droplet regions to Mt, accompanied by a fluorescence transition from green to red. This process corresponds to probe activation and the temporal transition from early lysosomal damage to subsequent mitochondrial damage 115. Similarly, SgTBB NPs initially accumulate in Mt and further migrate to the Nucleus after irradiation, inducing increased γ-H2AX expression and DNA oxidative damage (**Figure 9E**). The spatial changes in its near-infrared fluorescence signals can be used to track the mitochondrial–nuclear cascade damage process 19.

Compared with synchronous dual-organelle targeting, sequential therapeutic systems establish a clearer relationship among the processes of “initial damage—subcellular migration—secondary damage”, making them more suitable for using imaging information to determine therapeutic stages and optimize treatment timing. However, it should be noted that fluorescence conversion does not necessarily originate entirely from the therapeutic process itself, and may also be affected by probe metabolism, local polarity changes, or molecular aggregation states. Therefore, future studies should combine organelle functional response probes, time-resolved imaging techniques, and specific pathway inhibition experiments to further verify whether fluorescence changes truly reflect inter-organelle damage transmission rather than merely representing material redistribution. Sequential therapeutic platforms with long-wavelength emission characteristics and their ability for dynamic monitoring at the *in vivo* scale will be further discussed in the subsequent chapter on NIR-II fluorescence imaging.

#### 3.2.3 Imaging-guided combination therapy

Combination therapy strategies can partially overcome the limitations of single PDT caused by tumor hypoxia, antioxidant defense systems, and the restricted diffusion range of ROS. However, in multimodal therapeutic systems, imaging should not merely serve as a provider of fluorescence signals but should be used separately to evaluate delivery processes, monitor therapeutic parameters, and validate mechanisms of action. For example, MPTB integrates fluorescence imaging and photothermal imaging functions, where fluorescence signals confirm mitochondrial enrichment, while thermal imaging enables real-time monitoring of temperature changes during irradiation (**Figure 10A**). Through the synergistic effects of PDT and PTT, this platform can simultaneously disrupt mitochondrial function and promote ferroptosis and necroptosis 122. PTQ-TPA3 further integrates near-infrared fluorescence, photoacoustic imaging, and photothermal imaging (**Figure 10B**), providing cellular localization before treatment, tissue-scale distribution information, and temperature feedback during therapy, respectively. Its Type I PDT and PTT synergistically enhance lysosomal damage and further activate GSDMD-mediated pyroptosis and immunogenic cell death 116.

In addition to PDT/PTT combination systems, imaging technologies can also be applied to multifunctional platforms incorporating drug release or catalytic therapeutic modules. For example, Mn-ER-Cy utilizes near-infrared fluorescence and photoacoustic signals to track material delivery and therapeutic processes and enhances ER stress, induces pyroptosis, and promotes distant antitumor immune responses through the synergistic effects of ER-localized PDT, PTT, and Mn²⁺-mediated Catalytic Therapy (CDT) (**Figure 10C**) 126. NCB NPs-Pt/TAT monitors the acid-responsive nuclear delivery process through fluorescence imaging, followed by the synergistic induction of DNA damage through Pt (IV) prodrug release and PDT (**Figure 10D**), which further activates the cGAS-STING signaling pathway 127. However, for such combination therapeutic platforms, the independent contributions of different therapeutic modules—including irradiation, thermal effects, catalytic reactions, and drug release—still need to be separately elucidated to avoid inferring synergistic mechanisms solely based on overall tumor inhibition outcomes.

Therefore, the core of combination therapy lies in leveraging fluorescence imaging information to determine the initiation timing of distinct therapeutic processes, the targeted delivery of therapeutic agents, and whether the expected organelle damage has been achieved. Currently, most platforms can determine approximate irradiation times based on fluorescence signal changes or monitor temperature variations during treatment using thermal imaging; however, studies that further adjust light dosage, optimize treatment sequences, or establish treatment termination criteria based on real-time imaging feedback remain limited. Platforms that only achieve therapeutic localization and endpoint efficacy validation are more appropriately defined as “imaging-assisted therapy”. Only when imaging information directly participates in therapeutic parameter selection and treatment process regulation can the true value of “imaging-guided therapy” be realized.

### 3.3 Organelle-targeted therapy and immunogenic cell death

The significance of organelle-targeted therapy extends beyond the enhancement of localized cytotoxicity; it also lies in the ability to regulate downstream cell death pathways and immune outcomes by precisely selecting subcellular sites as the initial points of damage. Fluorescence imaging enables the visualization of therapeutic material delivery at the subcellular level, evaluation of the activation status of therapeutic components, and tracking of the transfer of therapeutic materials between multiple organelles. Nevertheless, imaging signals indicating the localization of therapeutic materials cannot be directly interpreted as evidence of organelle dysfunction or immune activation. Therefore, this chapter clarifies the evidence chain connecting organelle targeting, structural and functional organelle disruption, regulated cell death, and subsequent immune responses (**Figure 11**).

#### 3.3.1 Imaging-guided combination therapy

Different organelles are intricately linked to numerous cells signaling pathways, and local photodamage to organelles can trigger distinct cell death processes. Mitochondrial damage usually first manifests as a decrease in membrane potential, impaired electron transfer, and redox imbalance, followed by the activation of apoptosis, ferroptosis, or other cell death pathways. Lysosomal damage is characterized by LMP as a key event, which can amplify cytotoxicity through hydrolase release, autophagy dysfunction, and organelle cascade damage. Fluorescence imaging reveals the spatial localization of therapeutic materials and reflects the sequence of organelle damage to a certain extent, which subsequently aids in determining the specific cell death mode through corresponding molecular markers and functional assays.

ER damage is mainly manifested as protein folding stress, disruption of calcium homeostasis, and persistent ER stress, which can affect mitochondrial function and inflammatory cell death pathways through the UPR and calcium signaling. Nucleus-targeted therapy directly acts on genetic material, triggering cell death or innate immune signaling through oxidative DNA damage and double-strand breaks. Because a single platform often simultaneously generates ROS, thermal effects, and drug-induced cytotoxicity, it is necessary to integrate organelle functional changes, cell death pathway markers, and intervention-based blocking experiments to infer the occurrence of tumor cell apoptosis, ferroptosis, or pyroptosis.

#### 3.3.2 Immunogenic cell death and anti-tumor immunity

Immunogenic cell death (ICD) can convert local organelle damage into systemic anti-tumor immunity, with its typical process involving Calreticulin (CRT) exposure, High Mobility Group Box-1 Protein (HMGB1) release, and ATP secretion, followed by dendritic cell maturation and effector T cell recruitment. Different organelles can participate in this process at distinct regulatory nodes. Persistent oxidative damage in Mt can amplify intracellular stress and promote the release of danger signals. The mitochondrial network disruption, decreased membrane potential, and cristae damage induced by G-CQDs are associated with enhanced subsequent immune-related signaling 128. Lysosomal membrane permeabilization can amplify immune stimulation through hydrolytic enzyme release and Lysosome–Mt cascade damage, as demonstrated by M@P 113.

ER stress can promote CRT exposure and the release of other DAMPs; Ru1-induced ER damage is accompanied by activation of cGAS-STING and GSDMD-related pathways 123. Nuclear damage can generate cytosolic free DNA and activate the cGAS-STING pathway, and NCB NPs-Pt/TAT consequently links DNA damage with type I interferon signaling and T cell responses 127. It should be emphasized that fluorescence signals in these platforms primarily reflect material distribution, subcellular localization, or therapeutic activation status, whereas DAMP release, immune cell maturation, and infiltration are usually validated through independent endpoint assays. Therefore, “imaging-guided ICD” currently primarily refers to the use of imaging to optimize therapeutic implementation and establish associations with immune outcomes, rather than directly and continuously imaging immune responses themselves. Relevant NIR-II platforms and their applications in deep tissues will be discussed in subsequent sections.

Current studies remain insufficient to establish a complete evidence chain of “imaging–organelle damage–immune therapeutic efficacy”. First, colocalization coefficients or enhanced fluorescence intensity in tumor regions can only demonstrate that materials are close to target organelles or enriched in tumors but cannot directly quantify changes in membrane potential, LMP, ER stress, or DNA damage levels. Although some systems possess well-defined therapeutic mechanisms, their real-time imaging feedback remains limited. For example, Se-PC can enhance nuclear oxidative damage through DNA binding but lacks dynamic imaging of nuclear delivery and therapeutic progression 129. Second, material distribution imaging and immune indicator detection generally occur on different timescales: the former is primarily used to determine irradiation windows, whereas the latter is typically performed after treatment to evaluate CRT, HMGB1, ATP, and immune cell changes, with the relationship between them still primarily supported by correlation-based evidence.

When multiple effects, including PDT, PTT, CDT, and chemotherapy, coexist, it is also necessary to distinguish the independent contribution of specific organelle damage to cell death modes and immune activation. These studies suggest that future integrated theranostic research requires more comprehensive validation of subcellular localization, organelle functional alterations, cell death pathway activation, release of damage-associated molecular patterns (DAMPs), and systemic immune effects, while strengthening causal evidence through pathway inhibition experiments. Meanwhile, quantitative relationships should be established among imaging signals, therapeutic doses, functional damage thresholds, and immune response intensity. Most current platforms are still primarily validated in subcutaneous xenograft models, while deep orthotopic tumors, metastatic lesions, long-term metabolism, and organelle toxicity in normal tissues require systematic evaluation.

Overall, organelle targeting can concentrate therapeutic effects on key functional nodes of tumor cells, whereas imaging can be used to confirm therapeutic material delivery, determine whether therapeutic components are released or activated, select intervention timing, and evaluate treatment responses in combination with organelle functional indicators. Organelle-targeted activatable fluorescent probes hold great potential for the real-time monitoring of ICD hallmarks, including CRT exposure and HMGB1 release, as well as dynamic changes in ΔΨm. Such probes could serve as invaluable tools for the evaluation and optimization of IMT efficacy by providing functional feedback during treatment. Currently, most systems can achieve localization confirmation and treatment window selection, but functional imaging capable of directly reflecting membrane potential, LMP, ER stress, DNA damage, or immune activation levels remains limited. Therefore, theranostic integration should not be judged solely by whether a single material simultaneously possesses imaging and therapeutic capabilities but should instead consider whether imaging information actually contributes to determining treatment timing, dosage, sequence, or endpoints.

Due to tissue scattering, autofluorescence, and limited penetration depth, conventional fluorescence imaging faces substantial challenges in reaching deep-seated tumors *in vivo*. These constraints hinder the accurate assessment of therapeutic material delivery, tumor accumulation, and treatment timing. The integration of NIR-II fluorescence imaging into organelle-targeted theranostic platforms enhances the visualization of deep tumor localization, minimizes background interference, enables longitudinal tracking of probe distribution, and allows for the evaluation of tumor retention behavior, offering a promising solution to these challenges.

## 4. NIR-II fluorescence-guided organelle-targeted theranostic

NIR-II fluorescence imaging further enhances the performance of organelle-targeted theranostic platforms by enabling real-time monitoring of therapeutic material delivery in deep tissues. NIR-II (1000–1700 nm) fluorescence imaging can significantly reduce tissue scattering and background interference, thereby achieving greater imaging depth in living tumor models, enhancing the fluorescence intensity difference between tumor and normal tissues, and supporting longitudinal monitoring of biological processes 119. Importantly, NIR-II imaging can visualize the systemic distribution, tumor accumulation, and retention of therapeutic agents. These imaging advantages provide critical information for optimizing irradiation timing, selecting treatment windows, and evaluating therapeutic responses. Nevertheless, current *in vivo* NIR-II images primarily represent signals at the whole-body, organ, or tumor-region scale and cannot directly resolve individual organelles within deep tissues 130. Therefore, organelle localization and functional damage must still be validated through complementary methods, including cellular colocalization, time-resolved microscopy, and analyses of membrane potential changes, LMP, stress-related proteins, and DNA damage markers.

The core of NIR-II-guided organelle-targeted theranostics is to utilize NIR-II imaging for determining *in vivo* delivery behavior and treatment timing, while integrating cellular or in vitro validation to confirm the specific localization of organelles and their biological consequences. **Table 8** summarizes representative NIR-II fluorescence-assisted or fluorescence-guided organelle-targeted theranostic platforms.

### 4.1 NIR-II imaging-guided tumor localization and treatment window selection

NIR-II fluorescence imaging plays a pivotal role in organelle-targeted therapy by enabling continuous *in vivo* monitoring of therapeutic material delivery, including arrival at tumor sites, retention duration within tumor tissues, and the optimal timing for therapeutic intervention. It should be noted, however, that NIR-II imaging primarily provides tissue- and organ-level distribution information, rather than resolving localization at the organelle level. Thus, only by integrating *in vivo* pharmacokinetic data with cellular-level organelle localization and functional validation can one definitively demonstrate that a chosen treatment time point achieves both adequate tumor accumulation and effective intracellular delivery, yielding the expected therapeutic effects.

Ru1105 emits fluorescence at approximately 1105 nm under 808 nm excitation and is used to track the *in vivo* distribution of therapeutic agents and tumor accumulation, with approximately 48 h selected as the treatment time based on fluorescence signal changes. Due to its cationic and lipophilic properties, this material can distribute across organelles such as Mt, the ER, and Lysosomes, inducing multi-organelle oxidative damage upon irradiation 131.

The optimal imaging or irradiation times reported for different NIR-II theranostic platforms vary considerably. For example, approximately 6 h for LnNP@CUR-RGD 17, approximately 12 h for Indo3 and NPs Pt(II) metallacycles 133, 134, approximately 24 h for CM@ER-Rh NPs and TBTMQ 20, 132, approximately 24–48 h for IR-FCD-Ts NPs 135, and approximately 48 h for Ru1105. These differences stem from variations in material composition, particle size, administration routes, and tumor models, thus necessitating a comparative analysis of each theranostic platform regarding imaging/irradiation time, fluorescence intensity peaks, and maximum tumor-to-background ratio (TBR).

### 4.2 Sequential organelle targeting and intracellular trafficking

Some multi-organelle targeting systems undergo molecular restructuring, response activation, or location translocation after cellular uptake. For such platforms, it is crucial to determine the subcellular localization of the probe at different stages and whether their spatial transitions align with the temporal sequence of organelle damage and functional changes. NIR-II fluorescence imaging provides information on tumor accumulation, tissue distribution, and residence time; however, dynamic migration between organelles still requires verification through cellular time-lapse imaging, colocalization analysis, and functional biological assays. Therefore, when evaluating the efficacy of such theranostic platforms, changes in NIR-II fluorescence intensity within the tumor region cannot be directly interpreted as organelle migration; rather, tissue-level delivery information and subcellular functional processes must be clearly distinguished.

MPTB serves as a representative Mt-to-Nucleus sequential targeting photosensitizer. This AIE-type photosensitizer exhibits fluorescence emission at approximately 1072 nm, which is used to monitor tumor accumulation and assist in determining an irradiation time of approximately 24 h. Relevant cellular studies show that MPTB initially accumulates in Mt, inducing Type I ROS production upon irradiation and decreasing ΔΨm. Then MPTB migrates to the Nucleus, accompanied by oxidative DNA damage and activation of the cGAS-STING pathway. The evidence chain for this platform integrates *in vivo* tumor accumulation, dynamic migration observations at the cellular level, and functional indicators of membrane potential and DNA damage. It is worth noting that although the system's excitation efficiency and therapeutic penetration in deep tissues remain limited by the short excitation wavelength 132.

CM@ER-Rh NPs demonstrate a sequential damage process wherein ER stress propagates to mitochondrial dysfunction (**Figure 12A**). The system uses an approximately 1038 nm NIR-II phosphorescence signal to track the accumulation of materials in the tumor and selects approximately 24 h as the treatment time. Its ER-targeting structure and lipophilic cationic properties are conducive to intracellular distribution. After treatment, increased CHOP expression and enhanced eIF2α phosphorylation are observed initially, followed by a decrease in ΔΨm and mitochondrial dysfunction. These results support the transmission of ER stress to mitochondrial damage; however, the *in vivo* NIR-II signal mainly reflects the accumulation of materials within the tumor, and cannot directly distinguish localization changes between different organelles or report functional status in real time 20.

LnNP@CUR-RGD adopts an orthogonal excitation strategy to separate imaging from therapy (**Figure 12B–12C**). Under 808 nm excitation, it produces NIR-II fluorescence at approximately 1580 nm to monitor the biodistribution and tumor accumulation of nanoparticles; under 940 nm excitation, upconversion emission activates curcumin to generate singlet oxygen. The c(RGD-DPhe-K) ligand mediates initial binding to αvβ3 integrin, followed by transport to Lysosomes via endocytosis, with approximately 6 h selected as the therapeutic time window. This orthogonal design enables independent observation of material distribution and therapeutic activation; however, dynamic transport from the cell membrane to Lysosomes still requires cellular time-lapse imaging validation, and targeting specificity and energy conversion efficiency require further investigation 17.

During therapy, *in vivo* NIR-II imaging assists in determining the tumor accumulation process and the therapeutic time window, and reflects the spatial transfer of therapeutic materials between different organelles. Currently, the greatest contribution of NIR-II imaging remains its ability to provide *in vivo*-scale delivery and temporal information, thereby establishing a unified temporal reference for subsequent subcellular studies.

### 4.3 NIR-II imaging-guided combination therapy

The therapeutic effect of using PDT alone for tumor diagnosis and treatment is often limited by tumor hypoxia, antioxidant defenses, and the limited diffusion range of ROS. PDT is often combined with PTT, chemotherapy, or immunomodulation to achieve amplification of organelle damage via multiple signaling pathways. In these combination therapies, NIR-II imaging mainly monitors the accumulation of therapeutic agents in the body and determines the timing of intervention, so that different therapeutic components can act within the same spatial and temporal window. At present, NIR-II signals usually cannot independently visualize ROS generation, temperature changes, or drug release; therefore, their guiding function is still largely limited to the selection of treatment windows.

Indo3 NPs represent a lysosomal-targeted PDT/PTT combined treatment platform. Under 808 nm excitation, the platform emits fluorescence at about 1100 nm (**Figure 12D**); continuous imaging monitors tumor accumulation and determines a treatment window of about 12 h. After irradiation, ROS and photothermal effects jointly induce LMP, which then affects iron metabolism and mitochondrial function. Its weakly alkaline structure is protonated in acidic Lysosomes, enhancing intracellular retention, and the Pearson Correlation Coefficient (PCC) of cell colocalization analysis is 0.83, supporting it 133.

Pt (II) triangular metallacycles combine Type I PDT with chemotherapy (**Figure 12E**). This platform emits at approximately 1115 nm under 808 nm excitation and generates Type I ROS dominated by superoxide radicals (O₂•⁻). NIR-II signals are used to observe tumor distribution and assist in determining an approximately 12 h treatment time; the original study reported an imaging depth of approximately 7 mm. After internalization, the metallacycles preferentially accumulate in Lysosomes, where local ROS induces LMP and subsequent mitochondrial dysfunction; meanwhile, the Pt (II) component provides chemotherapeutic effects. Its lysosomal accumulation depends on physicochemical properties rather than specific active targeting ligands, and the complex synthesis process and long-term safety of platinum components require further evaluation 134.

IR-FCD-Ts NPs combine ER-targeted PDT/PTT with immune activation. This platform is excited at 1064 nm and emits fluorescence at 1273 nm (**Figure 12F**). NIR-II signals monitor tumor accumulation and retention, aiding in the selection of a 24–48 h therapeutic window. After irradiation, ER-localized ROS and photothermal effects disrupt protein homeostasis and induce CHOP-associated ER stress, promoting CRT exposure, HMGB1 release, and ATP secretion, which enhance dendritic cell maturation and CD8⁺ T cell infiltration. Notably, NIR-II imaging reflects material distribution and treatment timing, while ER stress and immune responses are validated through independent experiments; current evidence is primarily derived from subcutaneous tumor models 135.

Overall, NIR-II imaging provides essential *in vivo* distribution and temporal data for combination therapies, yet a real-time feedback mechanism for adjusting therapeutic parameters remains unestablished. Only when imaging results are actively used to select irradiation timing, adjust dosage, or arrange treatment sequences can the strategy be termed “NIR-II imaging-guided therapy”. If signals are used solely to observe tumor accumulation, the more appropriate term is “NIR-II imaging-assisted therapy”.

First, the spectral range of NIR-II platforms must be strictly defined, and reporting standards need further standardization. The emission peaks of MPTB, CM@ER-Rh NPs, Ru1105, Pt (II) metallacycles, Indo3 NPs, and LnNP@CUR-RGD all exceed 1000 nm; however, their excitation wavelengths are primarily 640, 660, or 808 nm. This represents the detection advantages of NIR-II emission rather than complete long-wavelength excitation–emission theranostic systems. IR-FCD-Ts NPs, with 1064/1273 nm excitation/emission, represent a relatively clear NIR-II excitation–emission system among the examples discussed in this chapter. Second, emission wavelength alone cannot substitute for comprehensive optical performance assessment. Studies should separately report excitation wavelength, emission peak, detection window, fluorescence quantum yield, molar extinction coefficient, brightness, photostability, signal-to-background ratio, penetration depth, and detector/filter information. Platforms such as MPTB have relatively low quantum yields; thus, short-wavelength NIR-II platforms emitting just beyond 1000 nm cannot fully represent the imaging performance across the entire 1000–1700 nm window. Only through side-by-side comparisons under identical conditions can the true *in vivo* advantages of long-wavelength emission be determined. Third, tissue-scale navigation and organelle localization require hierarchical validation. Current *in vivo* NIR-II images predominantly display overall accumulation in tumor regions, whereas organelle targeting evidence is obtained from cultured cells or *ex vivo* tissues. At present, most studies use superficial subepidermal tumor models to verify efficacy, and it is difficult to fully evaluate the actual role of NIR-II imaging in in situ tumors, deep organs, or tiny metastatic foci. Future studies should compare NIR-II imaging with NIR-I imaging in the same model, and the differences in signal-to-noise ratio, spatial resolution, minimum detectable foci size, and determination of treatment timing should be reported at various depths. Fourth, long-term safety and translational potential still require systematic evaluation. Ruthenium-, rhodium-, platinum-, and lanthanide-based nanoplatforms may exhibit long-term retention, slow clearance, and metal-associated toxicity; moreover, molecules with strong organelle affinity may continuously accumulate in normal tissues. Beyond short-term body weight monitoring and histological examination, evaluations should include long-term pharmacokinetics, excretion, immunotoxicity, repeated-dose effects, normal tissue function, and batch-to-batch consistency.

Future efforts should shift imaging signals from merely “observing accumulation” to “providing functional feedback”. Developing ratiometric, lifetime-based, or dual-channel NIR-II probes responsive to ROS, pH, GSH, membrane potential, LMP, or ER stress could directly link signals to therapeutic activation and organelle functional changes. Meanwhile, reproducible imaging thresholds and therapeutic rules should be predefined, enabling longitudinal imaging to guide adjustments in dosage, treatment sequence, or termination timing. Only through such strategies can we determine whether NIR-II provides merely clearer distribution images or truly delivers actionable information capable of improving therapeutic decision-making.

## 5. Challenges and Prospects

Organelle remodeling and functional dysregulation occur throughout tumor initiation, progression, metastasis, and therapeutic resistance, providing a rational basis for subcellular-scale tumor recognition and precise intervention. This review systematically summarizes five major organelles—Mt, Lysosomes, the ER, the Nucleus, and the GA—focusing on their targeting mechanisms, tumor-responsive activation strategies, and the applications of fluorescence imaging in PDT, combination therapy, immune regulation, and NIR-II imaging. In general, the organelle-targeted diagnosis and treatment platform has developed from the primary stage of simply pursuing organelle accumulation to an integrated system with multiple functions such as tumor-specific recognition, responsive activation, intracellular transport monitoring, and organelle damage regulation. The introduction of AIE materials, activated probes, ratiometric imaging, and NIR-II fluorescence technology has significantly improved the reliability of fluorescence signals, tumor differentiation ability, and *in vivo* monitoring level of the diagnosis and treatment system. However, how to convert cellular imaging information into quantitative and actionable evidence for therapeutic decision-making is still a core challenge in this field.

**The current organelle targeting strategy still needs to be further optimized.** Although the existing methods can basically achieve efficient organelle accumulation, there are still significant shortcomings in tumor selectivity and response to tumor heterogeneity. Mitochondrial targeting relies more on TPP, pyridine, and other lipophilic cations. Although such groups have efficient membrane penetration ability, their accumulation process depends on membrane potential, which may lead to non-specific distribution in normal tissues with active metabolism and reduce targeting stability after mitochondrial depolarization. The morpholine group and weakly alkaline amine used for lysosomal targeting mainly depend on the ion capture mechanism, but the acidic vesicles in normal cells can also cause non-specific retention. Targeted strategies for ER and Golgi bodies are mostly based on molecular identification elements such as SUR, COX-2, furin, and membrane environment, and their effects are easily interfered with by receptor heterogeneity and tumor diversity. Although nuclear localization sequences and DNA binding groups can enhance the efficiency of nuclear delivery, they face problems such as cell membrane barrier, non-specific genome interaction, and potential genetic toxicity. Therefore, simply increasing the number or affinity of targeted basic sequences is not the best strategy.

In the future, the design of the targeted platform should give priority to sequential identification strategies, that is, the tissue distribution should be limited by tumor-related signals first, and then controlled organelle localization and treatment activation should be realized. Specifically, tumor receptors, pathological microenvironment signals, or metabolic abnormalities can be used to regulate probe exposure, fluorescence activation, and therapeutic release by responding to enzymes, ROS, GSH, pH, hypoxia, or specific proteins. Such strategies help to reduce non-specific accumulation and improve the accuracy of diagnosis of different tumor types and disease stages. The comparative analysis in this review consistently shows that the multiple identification mechanism is usually better than the strategy of simply optimizing the classical targeting moieties in improving the ratio of tumor-to-normal tissue, signal-to-noise ratio, and treatment safety.

The second direction to be optimized is to expand the function of fluorescence imaging from monitoring material distribution to reporting organelle function status. At present, the fluorescence signals of most platforms are mainly used to present tumor accumulation, cell uptake, or organelle colocalization, while functional indicators such as ΔΨm loss, lysosomal membrane permeabilization, ERs, and DNA damage still need to be verified by independent biochemical detection or imaging methods. Although the above methods can confirm the positioning of the probe, they cannot directly determine whether the organelle damage has reached the effective treatment threshold, and it is difficult to guide the timing and intensity of treatment. Future research should focus on the development of reversible and ratiometric probes, and establish a quantitative correlation between imaging signals and functional biomarkers (including ΔΨm, LMP, ERs markers, and DNA damage indicators).

The construction of sequential multi-organelle cascade treatment strategy is one of the important directions for the development of diagnosis and treatment platforms in the future. Compared with the strategy of delivering multiple therapeutic components to multiple organelles at the same time, the sequential targeting mode relies on the timed utilization of specific organelle damage signals to trigger subsequent treatment events, so as to achieve a time-ordered treatment cascade response. Such cascade systems can break through the limitations of a single treatment method in terms of action and help reduce the non-specific systemic toxicity caused by the synchronous release of multiple components. However, the development of this kind of platform requires the establishment of a strict evidence chain system. At the living level, imaging research should verify the accumulation behavior of probes or drugs at the tumor site and the corresponding treatment time window; at the cell level, time-resolved imaging and functional detection need to jointly confirm the sequence of occurrence of different organelle damage and the timing relationship between them and the activation of downstream pathways. It should be pointed out that relying only on the change of fluorescence emission wavelength or the shift of the colocalization position of the probe is not enough to prove the signal transmission between organelles, because these changes may be influenced by factors such as the local polar environment, molecular aggregation state, or probe metabolism process, and need to be combined with functional indicators for comprehensive judgment.

The integration of organelle-targeted therapy and immune regulation is also an important development direction of diagnosis and treatment platforms. Cellular damage, including mitochondrial dysfunction, lysosomal membrane permeabilization, persistent ERs, and nuclear DNA damage, can promote the release of damage-related molecular patterns (DAMPs), induce inflammatory cell death, and activate an immune response, thus providing the conversion of local treatment to systemic anti-tumor immunity. The future platform design should not be limited to the simple superposition of different treatment methods, but should reasonably select complementary treatment strategies according to the biological vulnerability of target organelles. For example, by designing a variety of treatment modules that generate ROS, oxidative stress, nucleic acid damage, or ERs, the induction efficiency of immunogenic cell death can be enhanced. In the process of platform development, it is necessary to systematically evaluate the activation timing, individual contribution, and synergy of each treatment component. At the same time, sequential verification of organelle function changes, death pathway activation, DAMP release, immune cell maturation, and systemic immune response should be carried out to avoid simply attributing the complex coordination mechanism to the endpoint tumor inhibition effect.

The application of NIR-II fluorescence imaging should be further integrated into future organelle-targeted theranostic platforms. NIR-II imaging provides improved tissue penetration and signal-to-noise ratios, enabling dynamic monitoring of systemic distribution, tumor accumulation, and retention of therapeutic platforms, thereby supporting personalized treatment timing. Orthogonal excitation strategies may further separate imaging and therapeutic processes, reducing unintended activation of therapeutic modules by diagnostic illumination. However, current NIR-II *in vivo* images primarily provide information at the tissue or organ scale and cannot directly demonstrate specific organelle localization within deep tumor cells. Therefore, NIR-II-based theranostic platforms require multilevel validation combining *in vivo* pharmacokinetic imaging, cellular-scale localization analysis, and organelle functional assessment. Meanwhile, imaging parameters and performance metrics should be systematically reported and compared under standardized conditions, particularly when evaluating advantages over Near-Infrared I (NIR-I) imaging. For platforms using short-wavelength excitation but only entering the NIR-II window at the emission stage, their advantages should be clearly attributed primarily to detection capability rather than complete long-wavelength excitation and therapeutic performance.

Current studies provide a relatively clear design framework for next-generation theranostic platforms, integrating tumor selection, organelle localization, functional response, and therapeutic decision-making into a unified system. Tumor recognition improves selective accumulation, organelle targeting defines the subcellular site of action, functional responses provide measurable imaging feedback, and therapeutic decision-making enables treatment adjustment based on imaging information. Future platforms should prioritize structurally defined, simplified, and biodegradable materials to reduce synthetic complexity, batch variation, and unpredictable pharmacokinetic behavior caused by excessive functionalization. For metal complexes, rare-earth materials, and molecules with strong organelle affinity, systematic evaluation should include long-term retention, repeated administration, immune toxicity, normal tissue organelle function, and metabolic clearance, rather than relying solely on short-term body weight measurements and tissue sections for safety assessment. Moreover, clinically relevant models—including orthotopic tumors, deep organ tumors, metastatic lesions, patient-derived models, and clinical specimens—should gradually replace simplified subcutaneous tumor models to better evaluate tumor heterogeneity, target expression, response thresholds, and therapeutic windows.

In conclusion, the future development of organelle-targeted cancer theranostics will shift from constructing multifunctional systems that simply integrate imaging and therapy toward establishing spatiotemporally controlled theranostic platforms (characterized by a closed-loop mechanism with spatially precise targeting, dynamically optimized temporal windows, and imaging-feedback-driven therapeutic interventions) capable of guiding therapeutic decisions. Classical organelle-targeting motifs will continue to provide reliable delivery foundations, while activatable probes, AIE materials, ratiometric imaging, fluorescence lifetime imaging, and NIR-II technologies can enhance signal reliability, functional monitoring, and *in vivo* guidance. Sequential organelle damage and immune activation strategies may further improve therapeutic outcomes by establishing biologically regulated treatment cascades. Future progress will depend on the rational integration of these strategies with clear biological mechanisms, simplified material design, long-term safety evaluation, clinically relevant models, and standardized performance assessment.

## Figures and Tables

**Scheme 1:**
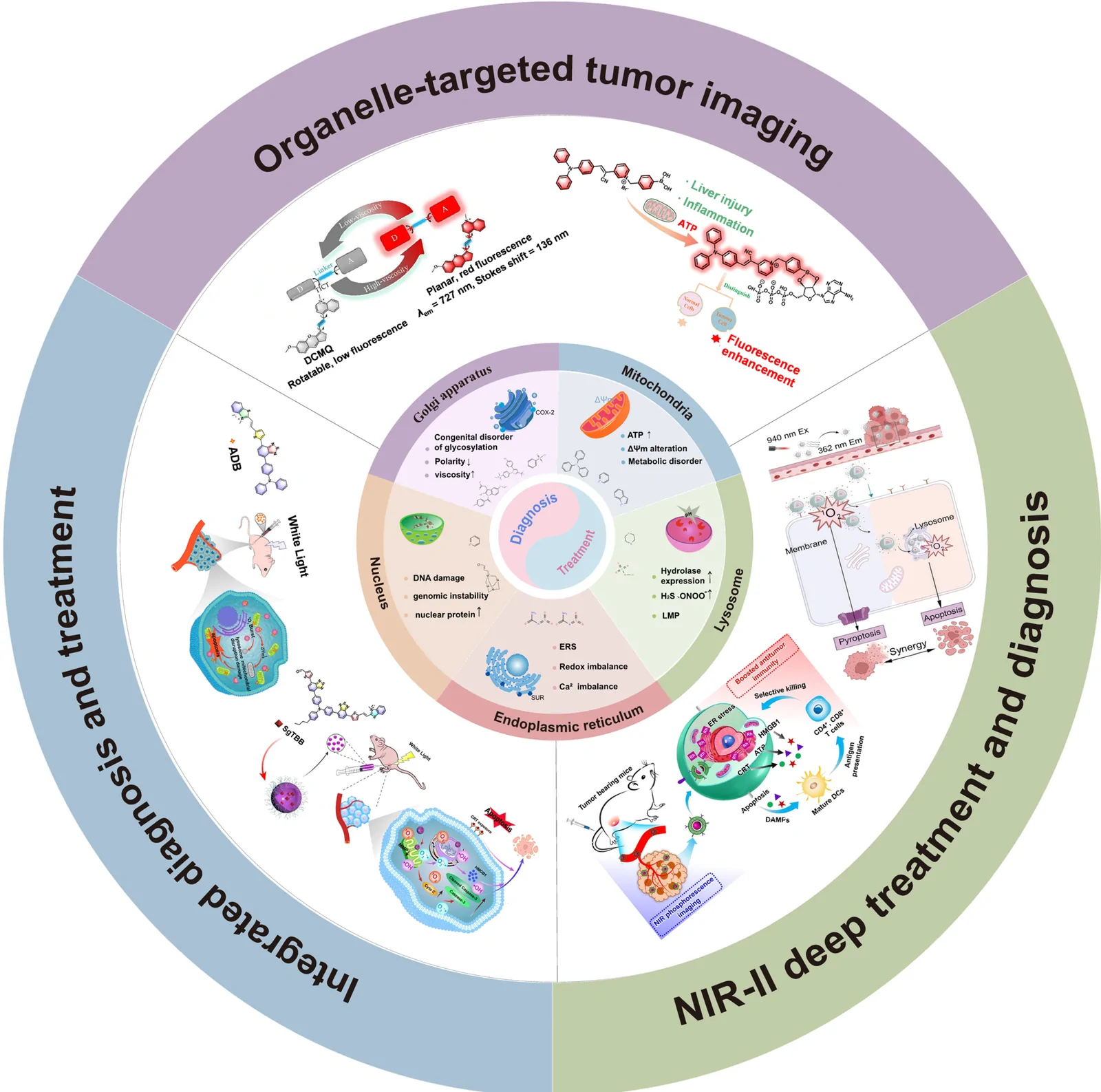
Schematic illustration of organelle-targeted diagnostic fluorescence imaging, integrated theranostic platforms, and Near-infrared II (NIR-II) imaging-guided cancer therapy, targeting five key organelles in cancer cells: Mitochondria, Lysosomes, Endoplasmic Reticulum, Nucleus, and Golgi apparatus. Adapted with permission from 17, copyright 2026 Elsevier. Adapted with permission from 18, copyright 2026 Elsevier. Adapted with permission from 19, copyright 2025 Elsevier. Adapted with permission from 20, copyright 2026 John Wiley & Sons. Adapted with permission from 21, copyright 2026 Elsevier. Adapted with permission from 22, copyright 2023 Elsevier.

**Figure 1:**
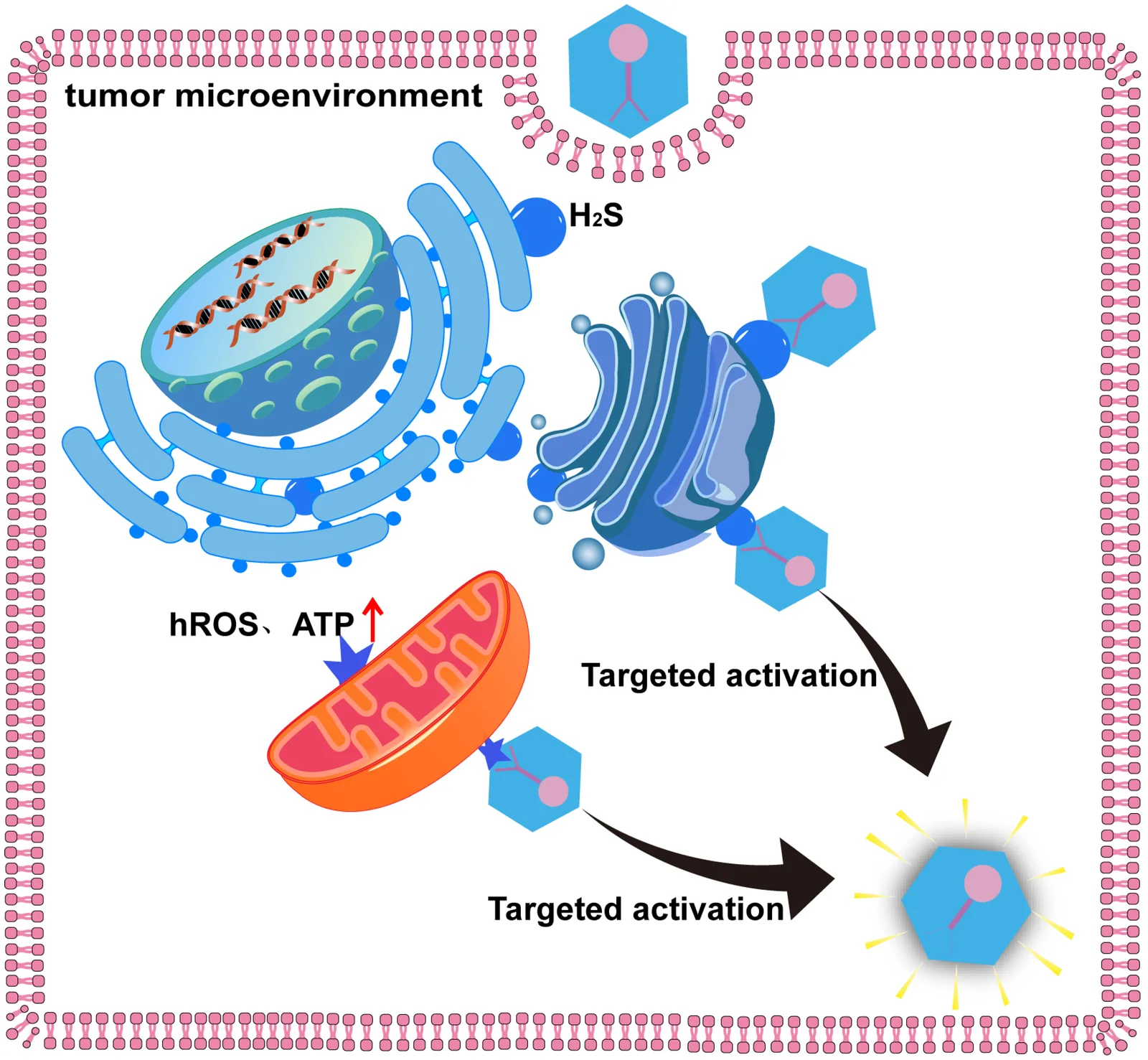
By integrating tumor microenvironment-responsive activation and specific metabolite recognition with canonical organelle-targeting strategies, can be designed more accurate organelle-targeted imaging platforms for cancer-selective imaging.

**Figure 2:**
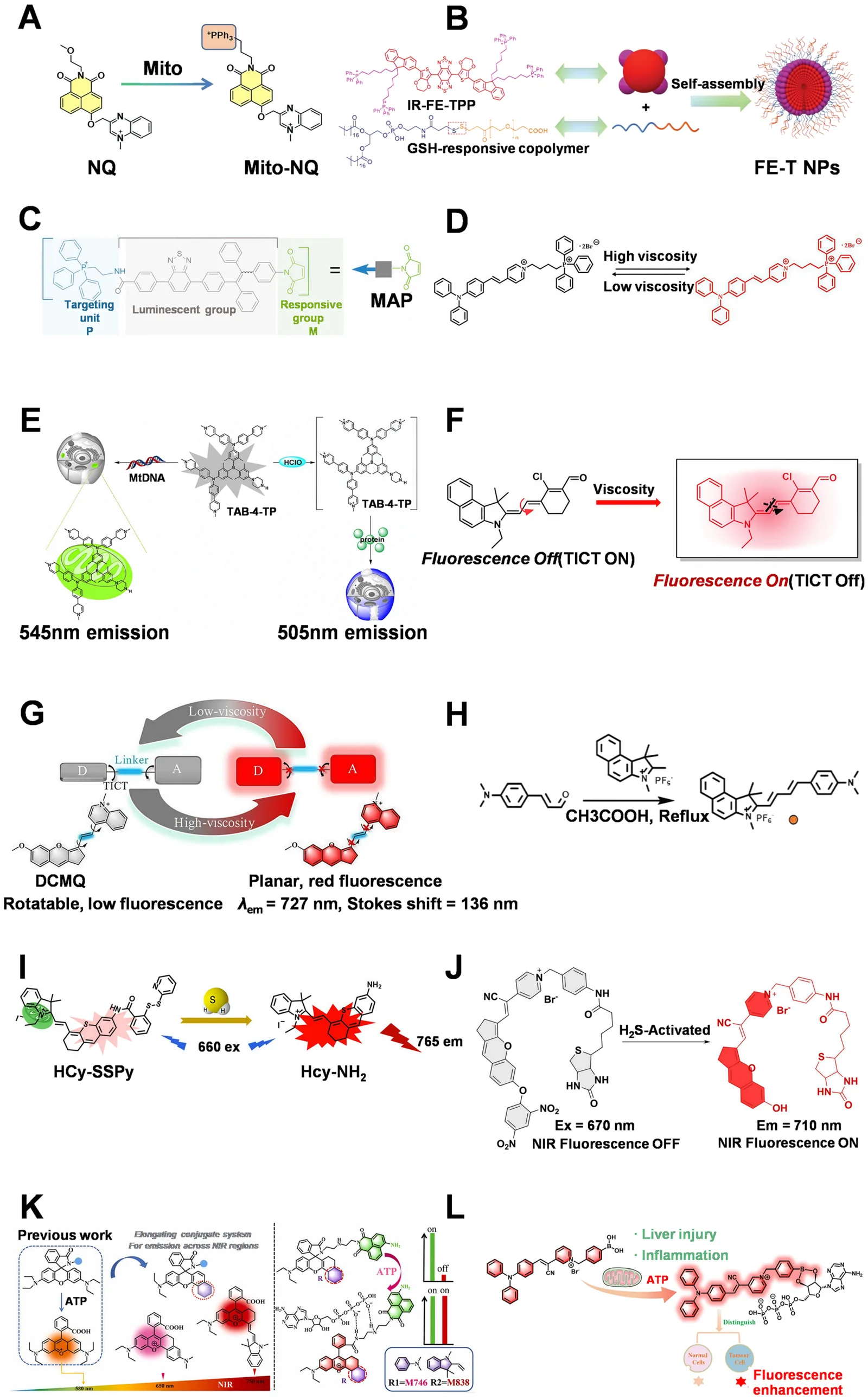
**Mt-targeted tumor diagnostic strategies. (A)** Mito-NQ introduced TPP for precise mitochondrial localization. Adapted with permission from 31, copyright 2026 American Chemical Society. (B) FE-T NPs were constructed through the self-assembly of a GSH-responsive copolymer and IR-FE-TPP. Adapted with permission from 32, copyright 2026 John Wiley & Sons. (C) Synthetic pathway of MAP. Adapted with permission from 34, copyright 2025 American Chemical Society. (D) TPA-P2 targeted Mt through TPP, with a schematic illustration of viscosity-activated fluorescence switching. Adapted with permission from 36, copyright 2026 Elsevier. (E) Dual-color fluorescence activation mechanism of TAB-4-Tp under different mitochondrial physiological states. Adapted with permission from 37, copyright 2026 Elsevier. (F) Fluorescence activation mechanism of Mito-BCy based on polarity responsiveness. Adapted with permission from 38, copyright 2026 American Chemical Society. (G) Viscosity-responsive mechanism of DCMQ based on the twisted intramolecular charge transfer (TICT) mechanism. Adapted with permission from 21, copyright 2026 Elsevier. (H) Chemical synthesis route of BHD and schematic illustration of its dynamic migration from Mt to Lysosomes. Adapted with permission from 39, copyright 2026 American Chemical Society. (I) Activation mechanism of HCy-SSPy in response to H₂S. Adapted with permission from 40, copyright 2025 American Chemical Society. (J) Chemical structure of 152T and schematic illustration of H₂S-specific activation. Adapted with permission from 41, copyright 2025 ROYAL SOCIETY OF CHEMISTRY. (K) M838 exhibited single-channel and dual-channel fluorescence responses through ATP recognition. Adapted with permission from 42, copyright 2026 Elsevier. (L) Fluorescence activation mechanism of TPA-P4 through ATP recognition. Adapted with permission from 18, copyright 2026 Elsevier.

**Figure 3:**
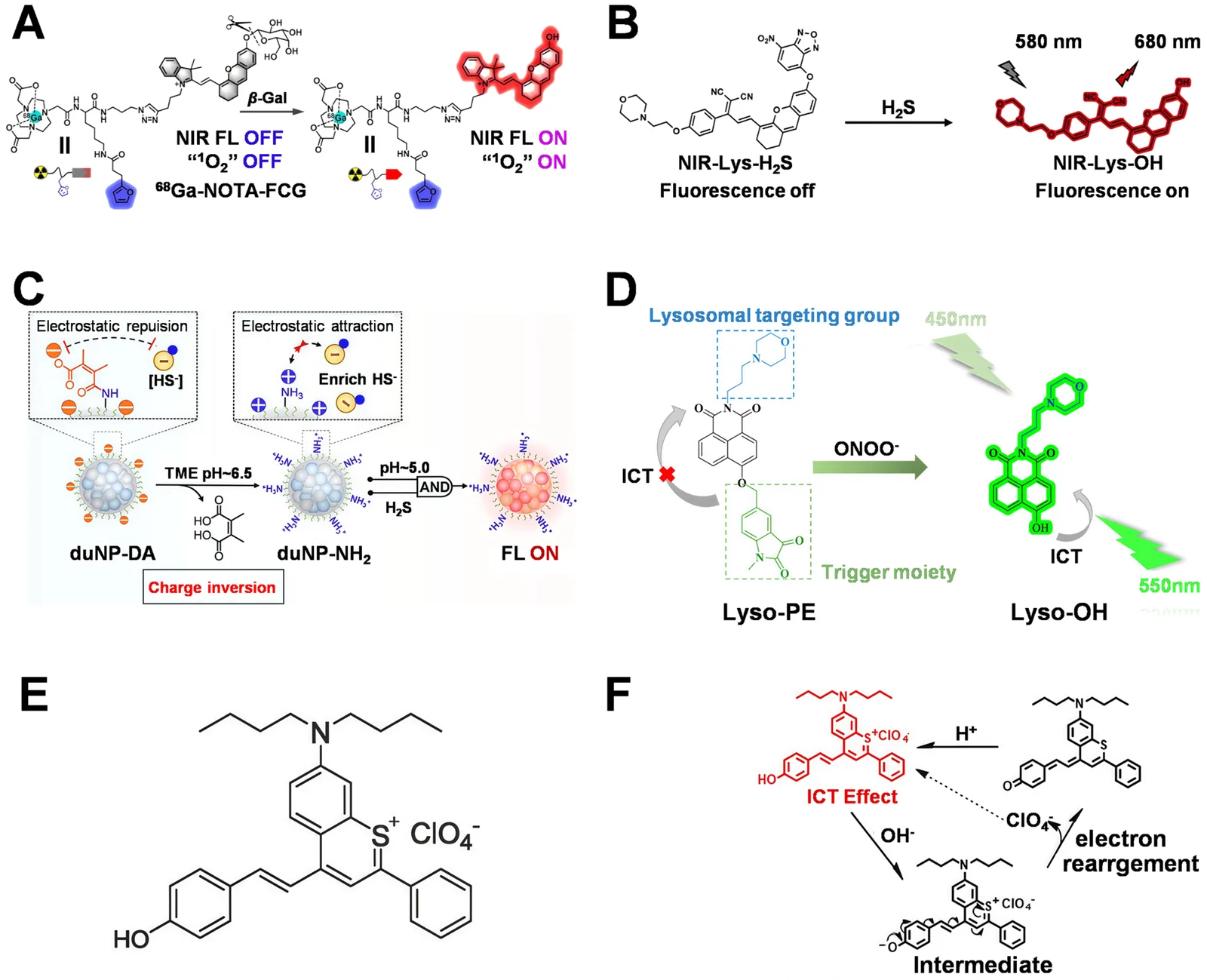
**Lysosome-targeted tumor diagnostic strategies.** (A) Schematic illustration of the β-gal-responsive mechanism of OELyso-Gal. Adapted with permission from 62, copyright 2026 American Chemical Society. (B) NIR-Lys-H₂S responds to H₂S by removing the NBD protecting group to generate NIR-Lys-OH. Adapted with permission from 63, copyright 2026 Elsevier. (C) duNP-DA undergoes charge reversal under the tumor microenvironment (TME, pH ~6.5), effectively enriches H₂S, and triggers fluorescence (FL) activation. Adapted with permission from 64, copyright 2026 American Chemical Society. (D) Schematic illustration of the ONOO⁻-specific “turn-off/turn-on” fluorescence response mechanism of Lyso-PE. Adapted with permission from 65, copyright 2026 Elsevier. (E) Chemical structure of SOH. Adapted with permission from 66, copyright 2026 Elsevier. (F) Schematic illustration of the pH-responsive tautomerization mechanism of SOH based on sulfonium/thiopyrylium salt dyes. Adapted with permission from 66, copyright 2026 Elsevier.

**Figure 4:**
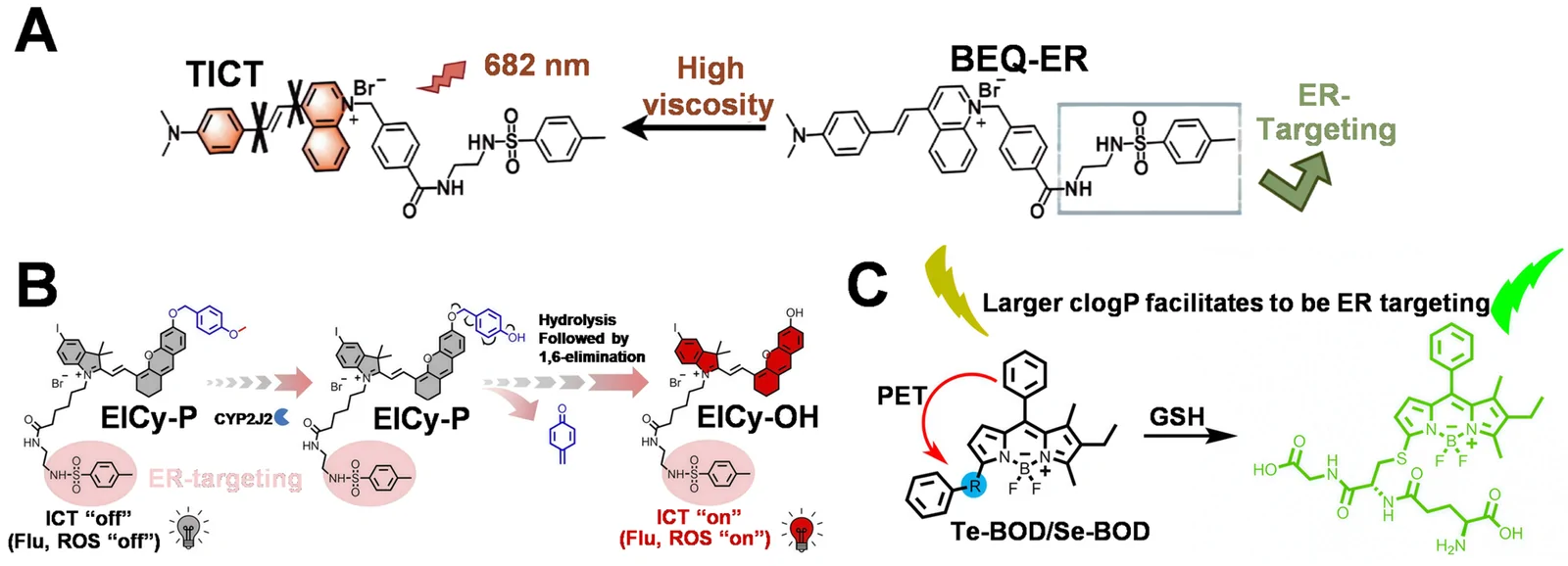
**ER-targeted tumor diagnostic strategies.** (A) Fluorescence “turn-on” response mechanism of BEQ-ER under high-viscosity conditions. Adapted with permission from 79, copyright 2026 Elsevier. (B) Schematic illustration of the “dual photosensitizer” response mechanism of EICy-P, involving deprotection under CYP2J2 catalysis to activate fluorescence (Flu) and ROS generation. Adapted with permission from 80, copyright 2026 Elsevier. (C) Fluorescence activation mechanism of Te-BOD/Se-BOD probes through GSH-responsive inhibition of the PET effect. Adapted with permission from 81, copyright 2026 Elsevier.

**Figure 5:**
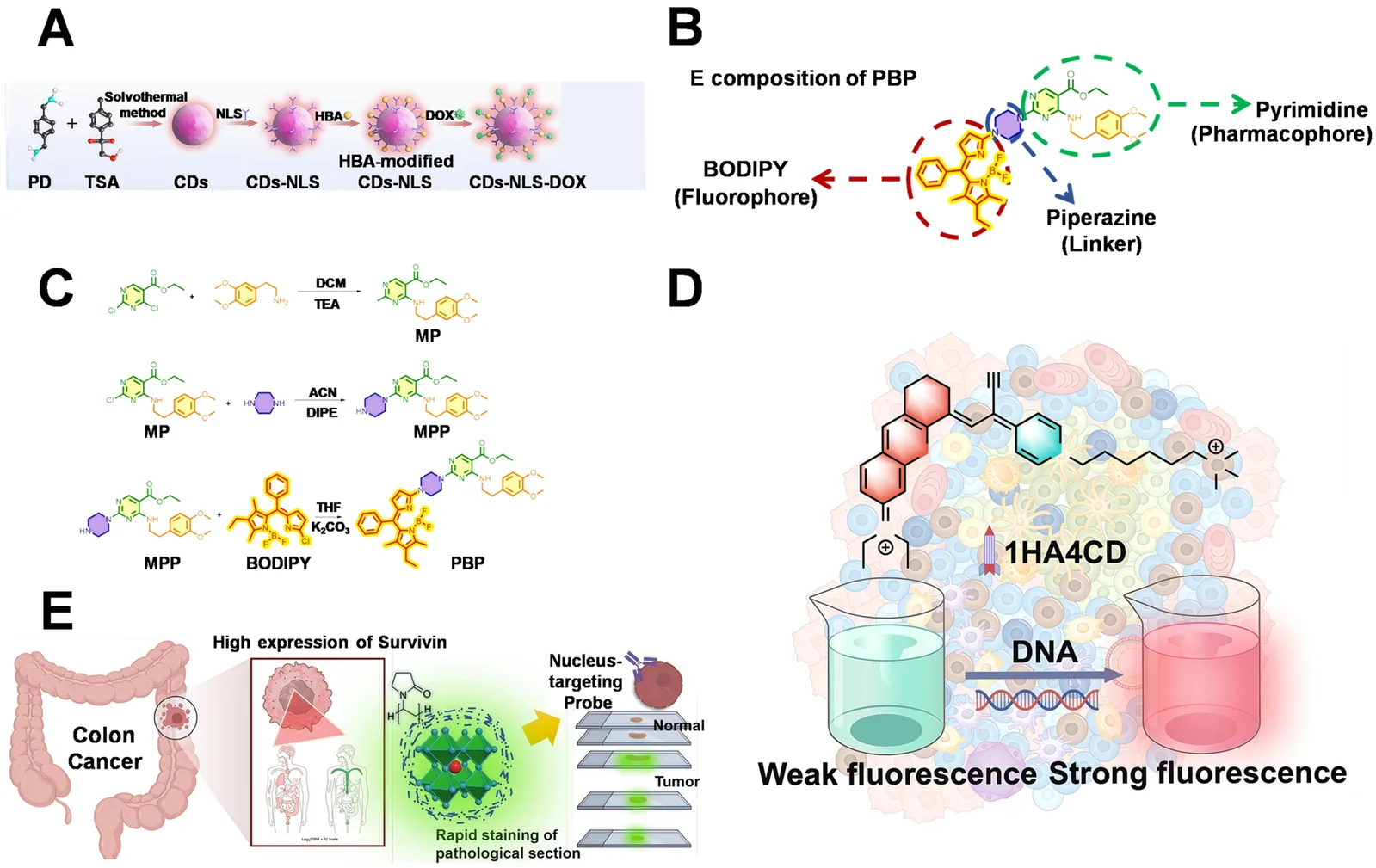
** Nuclear-targeted tumor diagnostic strategies.** (A) Synthetic route of CDs-NLS-DOX. Adapted with permission from 93, copyright 2026 Elsevier. (B) Structural interpretation of different components of the DSPC-PBP probe. Adapted with permission from 90, copyright 2026 Elsevier. (C) Chemical synthesis route of DSPC-PBP through a three-step modular reaction. Adapted with permission from 90, copyright 2026 Elsevier. (D) Fluorescence “turn-on” response mechanism of 1HA4CD triggered by DNA binding. Adapted with permission from 91, copyright 2026 Elsevier. (E) Schematic illustration of efficient tumor tissue discrimination using the nuclear-targeted fluorescent probe PVP@PNCs@Ab through rapid pathological section staining. Adapted with permission from 92, copyright 2026 Elsevier.

**Figure 6:**
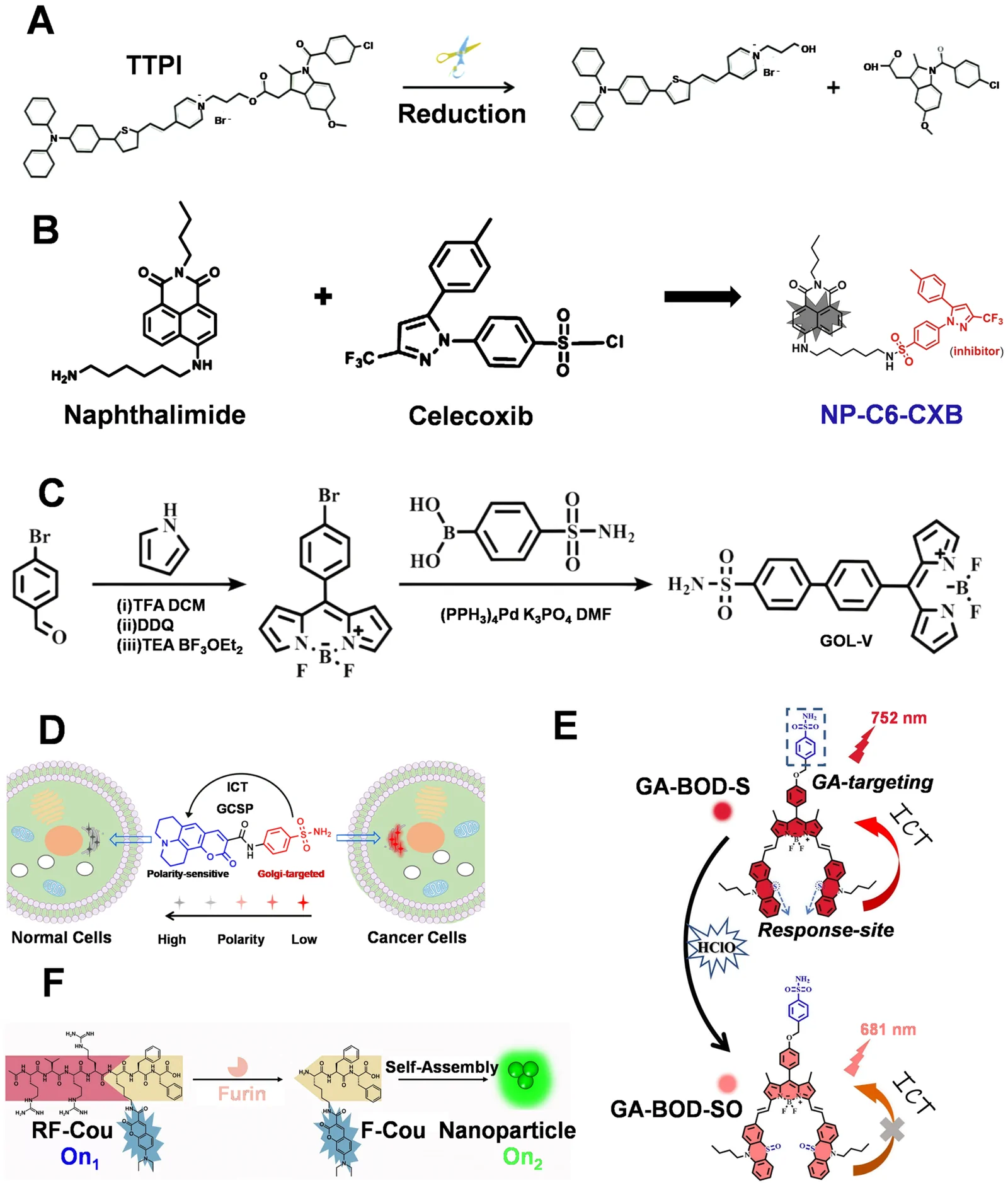
**GA-targeted tumor diagnostic strategies.** (A) Schematic illustration of TTPI structural composition. Adapted with permission from 97, copyright 2026 Elsevier. (B) Schematic illustration of NP-C6-CXB synthesis. Adapted with permission from 98, copyright 2026 American Chemical Society. (C) Chemical synthesis route of GOL-V and schematic illustration of viscosity-responsive fluorescence activation. Adapted with permission from 99, copyright 2026 Elsevier. (D) GCSP responds to differences in GA microenvironment polarity and enables fluorescence imaging of tumor cells. Adapted with permission from 100, copyright 2026 Elsevier. (E) Mechanism of GA-BOD-S fluorescence emission blue shift induced by HClO-specific oxidation. Adapted with permission from 101, copyright 2026 Elsevier. (F) Mechanism of RF-Cou fluorescence activation through specific Furin response. Adapted with permission from 102, copyright 2026 Elsevier.

**Figure 7:**
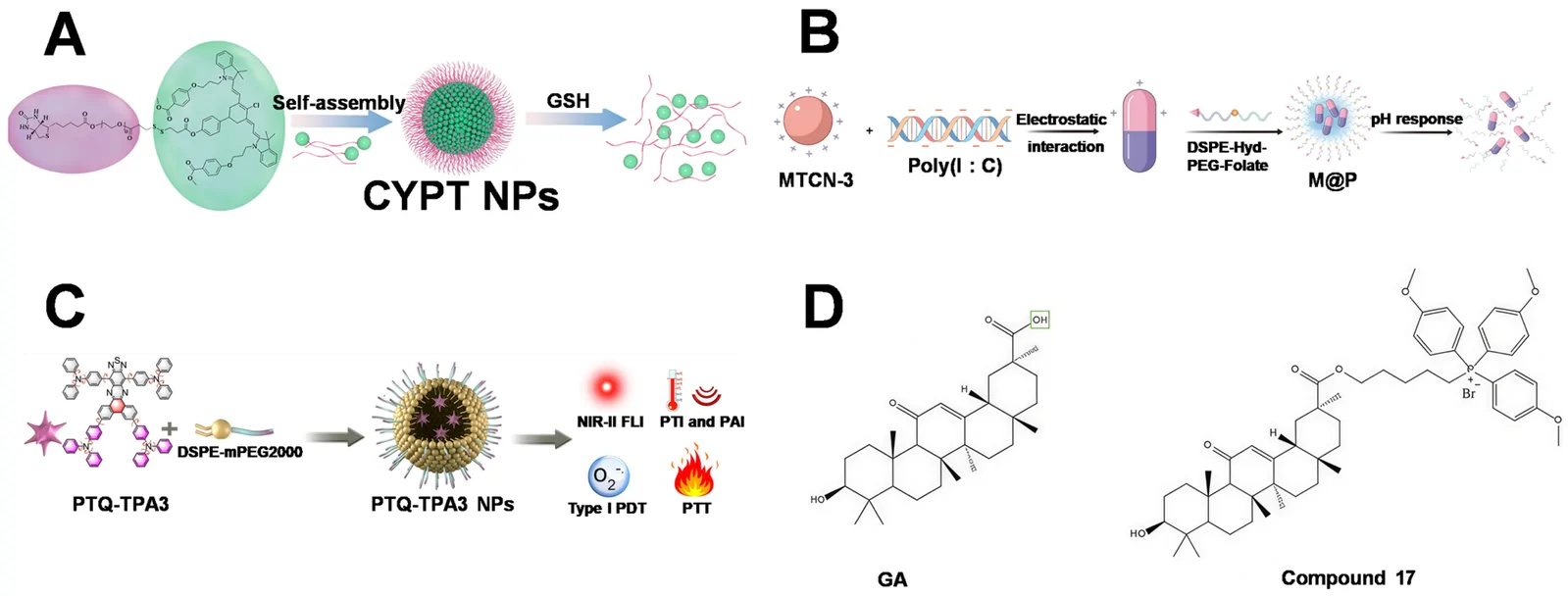
**Fluorescence imaging-guided cancer therapy strategies.** (A) Schematic illustration of the self-assembled structure of CYPT NPs and the GSH-responsive disassembly of CYPT NPs. Adapted with permission from 112, copyright 2026 Elsevier. (B) Synthetic route of M@P and schematic illustration of responsive release under acidic microenvironment conditions. Adapted with permission from 113, copyright 2025 Royal Society of Chemistry. (C) Schematic illustration of the self-assembly of PTQ-TPA3 NPs and their multimodal imaging-guided type I photodynamic/photothermal synergistic theranostics functions. Adapted with permission from 116, copyright 2026 Elsevier. (D) Schematic illustration of the structure of Compound 17. Adapted with permission from 118, copyright 2026 MDPI.

**Figure 8:**
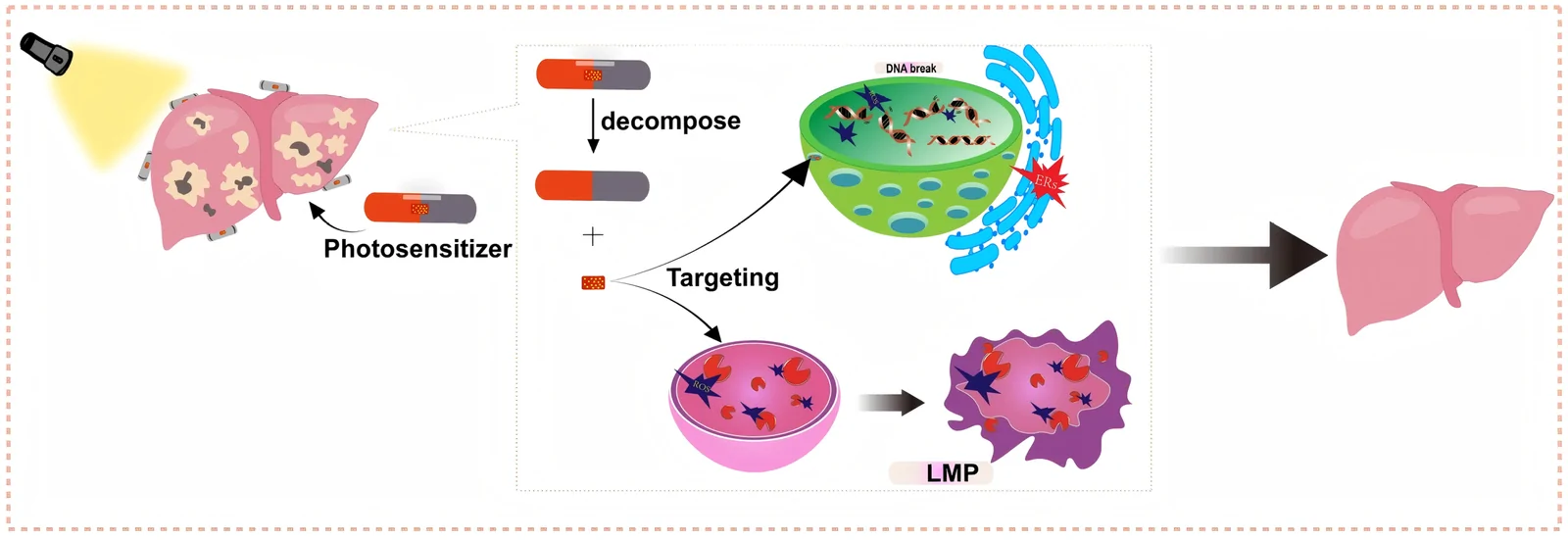
Organelle-targeted PDT for tumor treatment.

**Figure 9:**
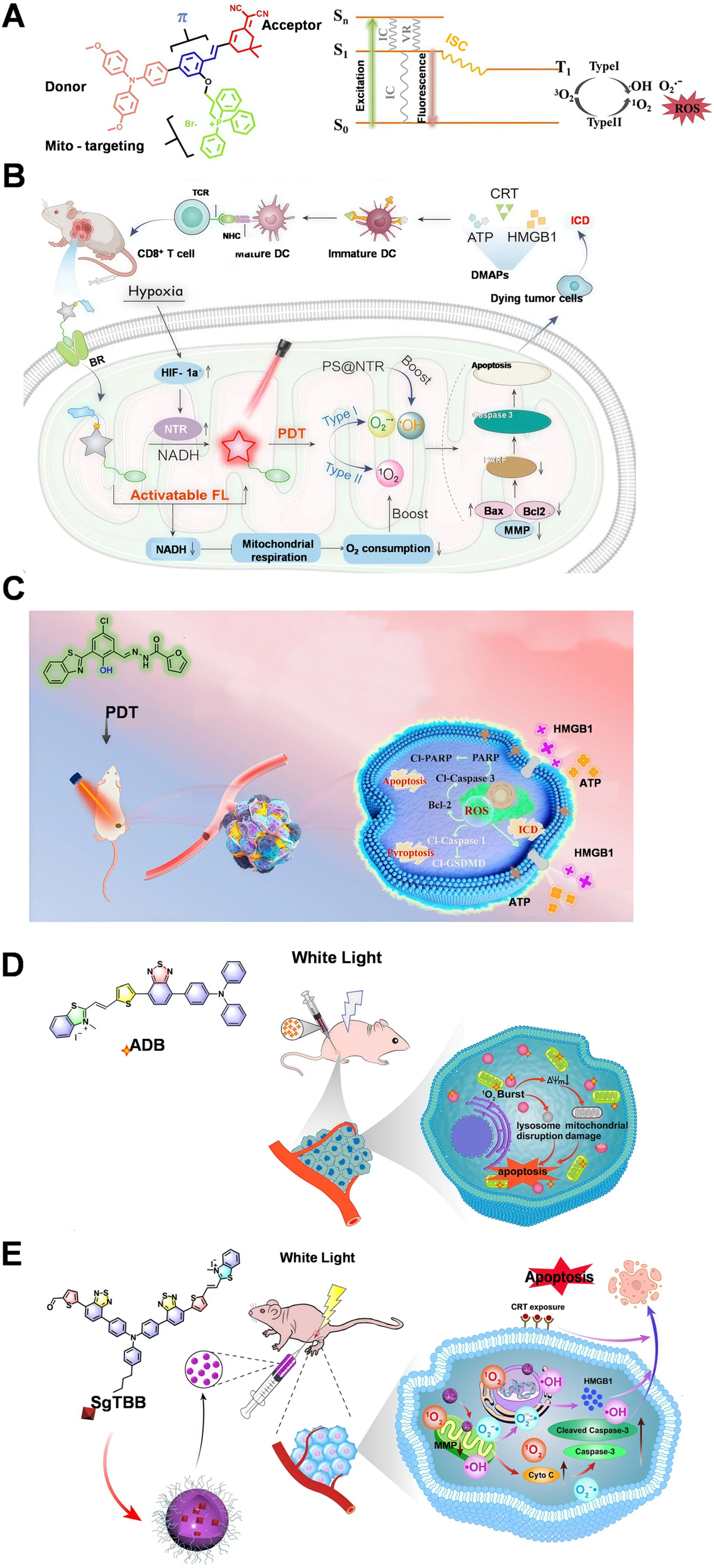
**Organelle-targetedPDT-1.** (A) Schematic illustration of the photophysical mechanism by which Mito-IsTp efficiently generates ROS through Type I and Type II pathways. Adapted with permission from 120, copyright 2026 Elsevier. (B) Schematic illustration of NO₂/BDP-BT for enhanced PDT efficacy and amplified anti-tumor immune responses. Adapted with permission from 121, copyright 2026 Elsevier. (C) Chemical structure of compound 3 and its PDT therapeutic mechanism. Adapted with permission from 124, copyright 2026 Elsevier. (D) Chemical structure and mechanism of action of ADB. Adapted with permission from 22, copyright 2023 Elsevier. (E) Chemical structure of SgTBB NPs and its tumor therapeutic mechanism. Adapted with permission from 19, copyright 2025 Elsevier.

**Figure 10:**
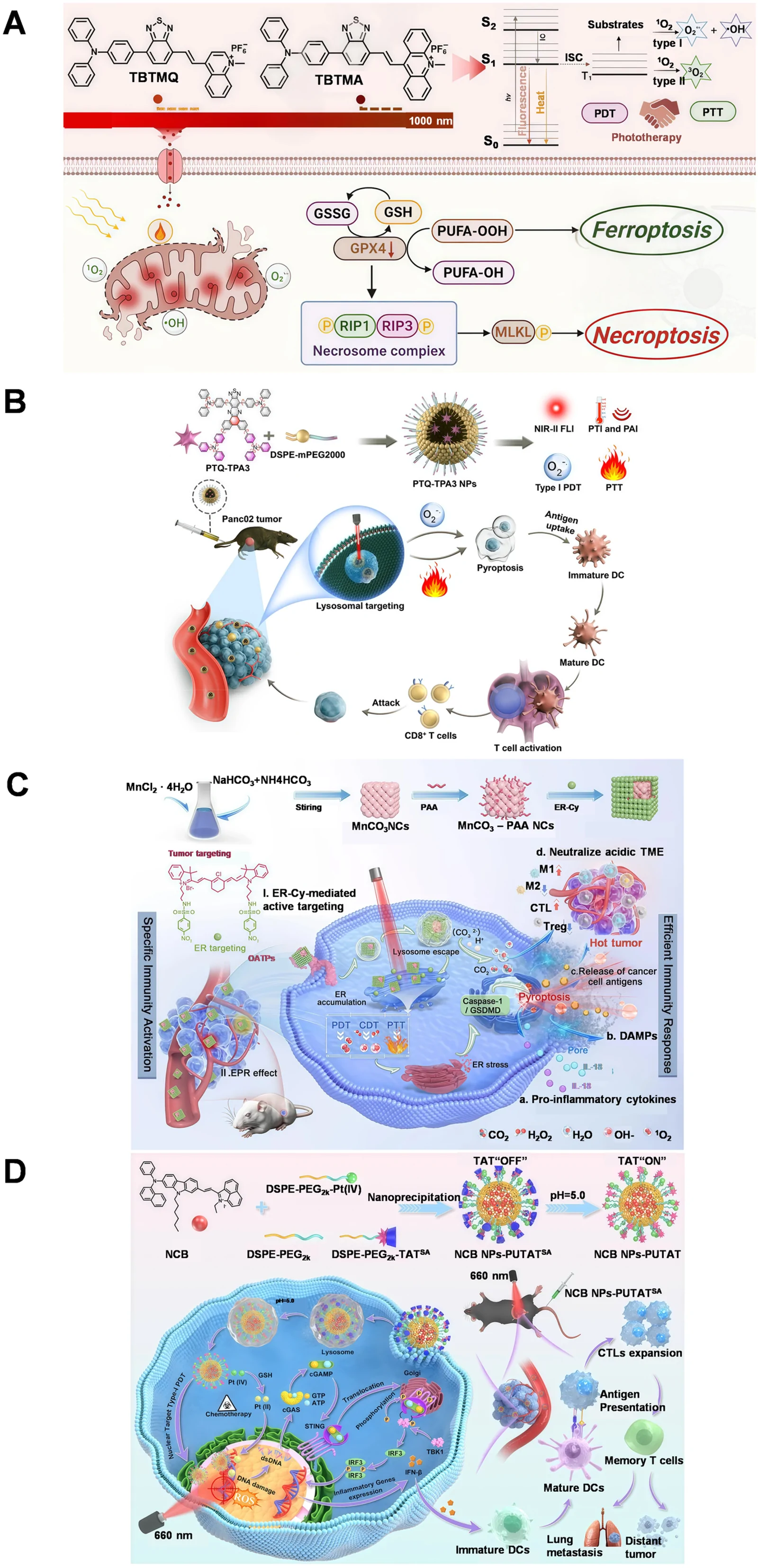
**Organelle-targeted PDT-2.** (A) MPTB targets Mt under near-infrared light excitation and triggers ferroptosis and necroptosis through synergistic photothermal / photodynamic effects. Adapted with permission from 122, copyright 2026 John Wiley & Sons. (B) PTQ-TPA3 integrates near-infrared fluorescence, photoacoustic imaging, and photothermal imaging to treat tumors through lysosomal damage-mediated mechanisms. Adapted with permission from 116, copyright 2026 Elsevier. (C) Synthetic route of Mn-ER-Cy and schematic illustration of anti-tumor immunity achieved through PDT/CDT/PTT synergistic effects. Adapted with permission from 126, copyright 2025 Springer Nature. (D) NCB NPs-Pt/TAT triggers Nucleus-targeted Type I PDT and platinum chemotherapy synergy under 660 nm irradiation to induce anti-tumor immune responses. Adapted with permission from 127, copyright 2026 Elsevier.

**Figure 11:**
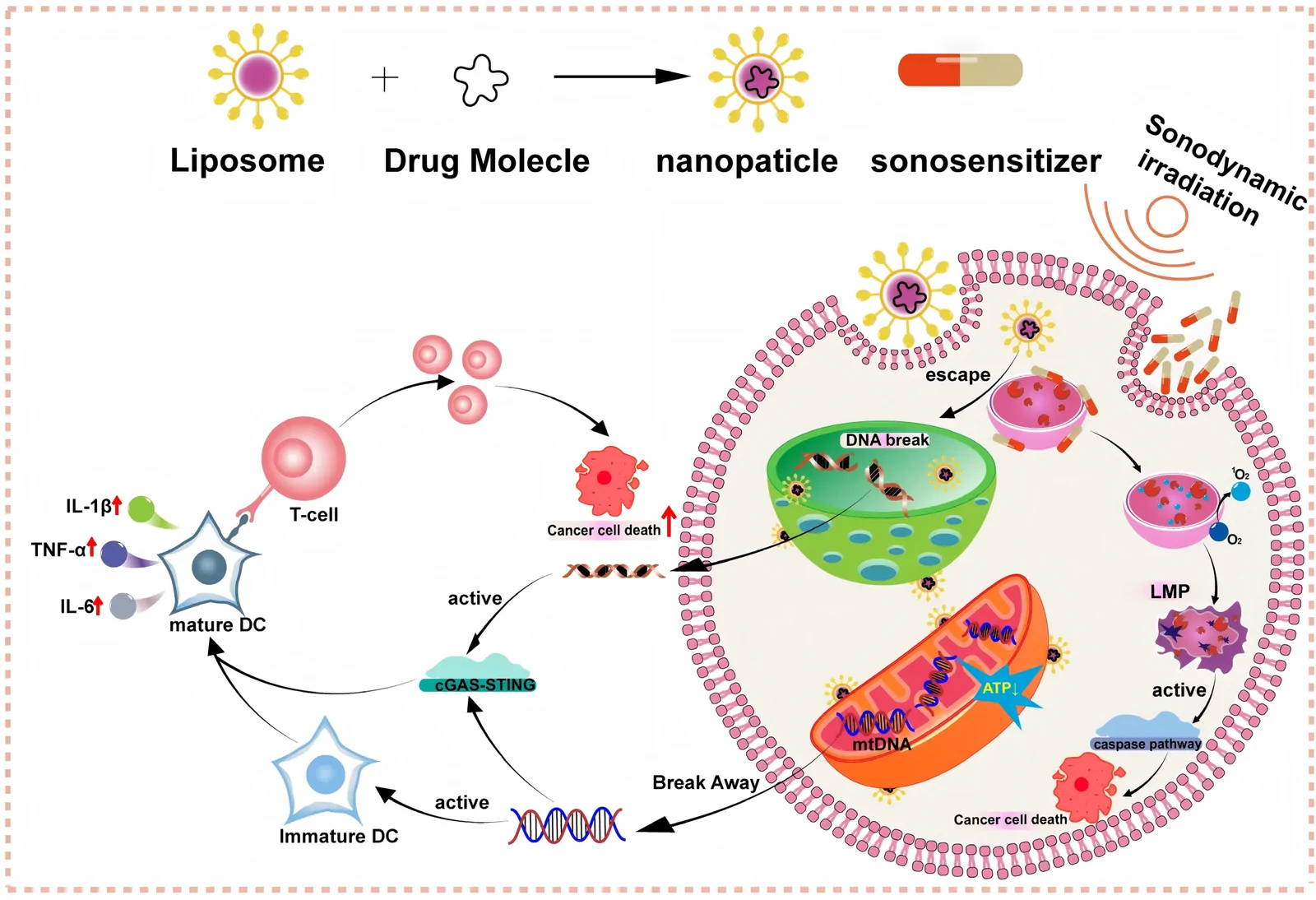
The synergy of PDT, sonodynamic therapy (SDT), and IMT achieves highly effective antitumor treatment.

**Figure 12:**
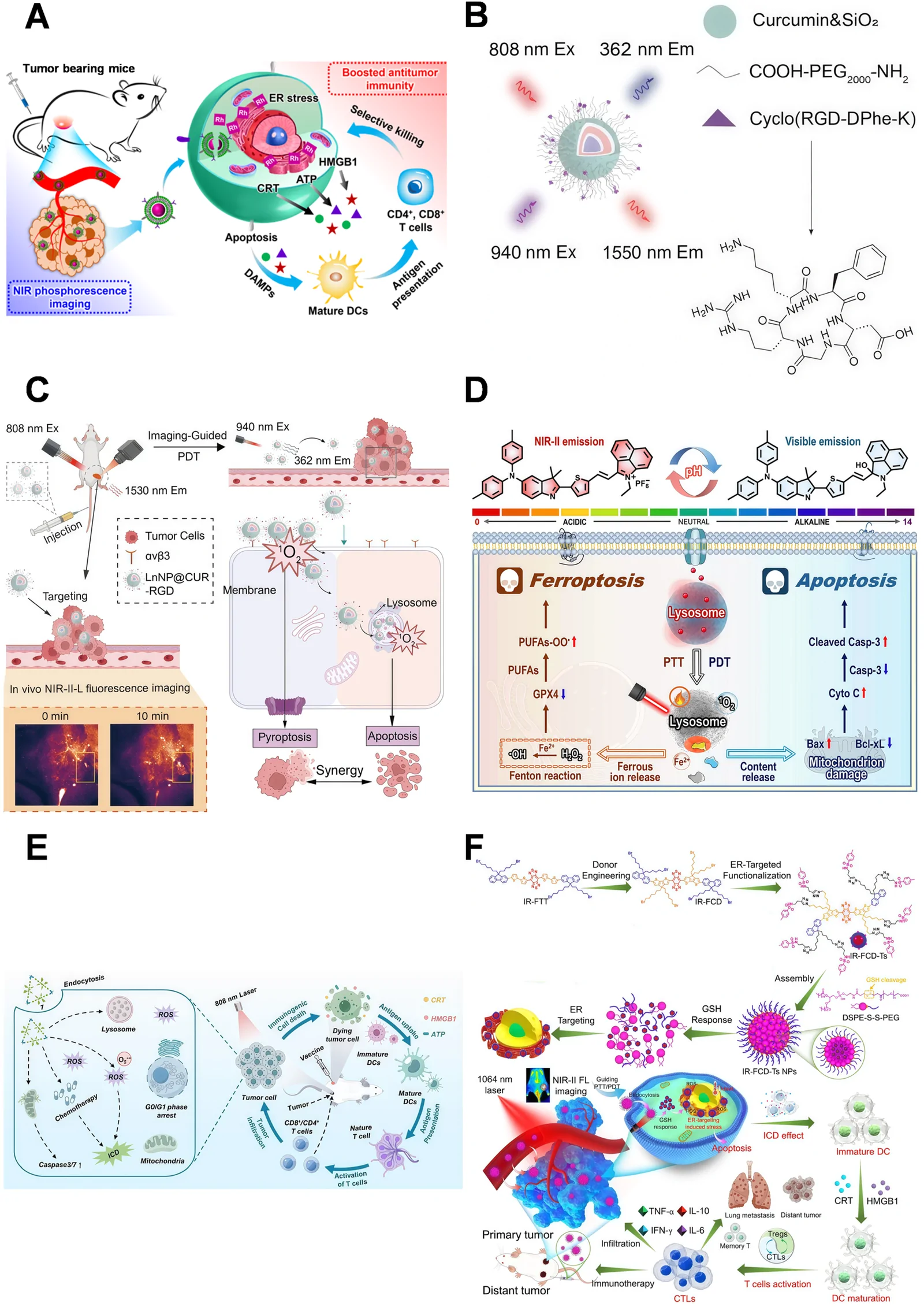
** NIR-II fluorescence-guided organelle-targeted theranostics.** (A) Schematic illustration of the tumor therapeutic mechanism of CM@ER-Rh NPs. Adapted with permission from 20, copyright 2026 John Wiley & Sons. (B) Structural design of LnNP@CUR-RGD. Copyright 2026 Elsevier. Adapted with permission from ref. Adapted with permission from 17, copyright 2026 Elsevier. (C) Schematic illustration of the phototheranostic mechanism of LnNP@CUR-RGD. Adapted with permission from 17, copyright 2026 Elsevier. (D) Schematic illustration of the pH-responsive fluorescence activation mechanism of Indo3 NPs and tumor killing through lysosomal disruption-mediated ferroptosis and apoptosis via PTT/PDT. Adapted with permission from 133, copyright 2026 Elsevier. (E) Schematic illustration of the immunotherapeutic mechanism of Pt (II) triangular metallacycles, which enter cells through endocytosis and induce ICD and DAMP release through light-triggered ROS generation and chemotherapy synergy. Adapted with permission from 134, copyright 2026 John Wiley & Sons. (F) Synthetic route of IR-FCD-Ts and schematic illustration of the mechanism by which IR-FCD-Ts achieve GSH-responsive and ER-targeted photothermal/photodynamic synergistic therapy under NIR-II fluorescence imaging guidance to induce ICD and effectively inhibit tumors. Adapted with permission from 135, copyright 2025 Springer Nature.

**Table 1:** Typical targeting motifs for organelle-targeted cancer imaging platforms.

Organelle	Biological basis	Targeting motif	Tumor selectivity	Advantages	limitations
GA	Altered trafficking, glycosylation and tumor-associated metabolism	COX-2 ligands, benzenesulfonamides, Furin-responsive peptides	Protein/enzyme recognition or microenvironment-responsive activation	Useful for functional imaging and specimen analysis	Few validated targeting motifs; limited deep-tissue evidence
Mt	Elevated membrane potential and altered metabolism	TPP, pyridinium, indolium, quinolinium	Tumor-responsive activation or secondary mitochondrial recognition	Efficient uptake and broad probe compatibility	Accumulation in normal Mt; sensitivity to depolarization
Nucleus	Nuclear transport and DNA/protein interactions	NLS, TAT peptides, DNA-binding ligands, protein-recognition motifs	Tumor-associated nuclear protein recognition or conditional activation	Direct access to genomic stress and drug delivery	Delivery barriers, normal-DNA binding and genotoxicity
Lysosome	Acidic lumen and lysosomal enzyme activity	Morpholine, weak bases, sulfonium, enzyme-responsive motifs	Acid trapping combined with tumor-associated enzyme or metabolite activation	Simple and well-established retention mechanism	Acidic normal organelles can cause off-target accumulation
ER	Membrane composition, protein folding and stress responses	p-Toluenesulfonamide, sulfonamides, lipophilic or bile-acid-derived groups	Receptor/membrane affinity combined with tumor-responsive activation	Supports functional ER-stress imaging and therapy	Limited validated motifs and receptor heterogeneity

Abbreviations: GA, Golgi apparatus; Mt, Mitochondrion; ER, Endoplasmic Reticulum; COX-2, Cyclooxygenase-2; TPP, Triphenylphosphine; TAT, Trans-Activator of Transcription; NLS, Nuclear localization sequence.

**Table 2:** Representative Mt-targeted cancer imaging platforms classified by functional motifs.

Probe	Functional motif	Ex/Em (nm)	T/N	Key feature	Limitation	Ref.
Mito-NQ	TPP	450/550	3.0	Tumor boundary identification; 3D tumor imaging	Long response time (70 min); insufficient biodistribution and safety evaluation	31
FE-T NPs	TPP	808/1000-1400	2.84	Deep-tissue imaging; high signal-to-noise ratio; phototherapy integration	Always-on fluorescence; complex preparation	32
SRDN	TPP	630/665	2.16	Signal amplification; enhanced mitochondrial accumulation	Limited tissue penetration; intracellular degradation risk	33
MAP	TPP	405/600	9.5	Dual mitochondrial anchoring; surgical guidance	Long response time (4 h); mtUPR expression may vary under stress conditions	34
TPNP	Pyridinium	TP 470/5754OP 860/575		Deep-tissue 3D imaging; tumor-selective activation	Unclear *in vivo* toxicity and targeting efficiency	35
TPAP2	TPP, Pyridinium	470/600		Rapid targeting; wash-free imaging; enhanced fluorescence	Increased nonspecific interaction with negatively charged organelles	36
TAB-4-TP	Pyridinium	420/525		Dual recognition of ROS and mtDNA	Limited *in vivo* validation	37
Mito-BCy	Quaternary ammonium	490/635	3.5	Mitochondrial microenvironment monitoring; tumor imaging	Lack of active targeting strategy; insufficient quantitative colocalization analysis	38
DCMQ	Quinoline cation	591/727		High signal-to-noise ratio; *in vivo* tumor imaging	Low quantum yield; limited pharmacokinetic and safety data	21
BHD	Quaternary ammonium	624/720		Mt–Nucleus dual targeting; enhanced fluorescence	Potential nonspecific electrostatic interactions	39
HCy-SSPy	hemicyanine	660/765		Rapid H₂S response and deep-tissue imaging	Long-term toxicity requires evaluation	40
152T	biotin	680/710		High selectivity and tumor retention	Limited application in deep tumors	41
M838	rhodamine–cyanine hybrid scaffold	480/749	4.0~4.4	First reversible NIR ratiometric ATP imaging probe; dynamic monitoring	Primarily suitable for high ATP levels	42
TPA-P4	Pyridinium	450-620	2.0~2.5	AIE-enhanced ATP detection and mitochondrial imaging	NIR-I emission; potential biological interference	18

Abbreviations: Ex/Em, Ex/Emission; T/N, Tumor-to-Normal ratio; TPP, Triphenylphosphine; ATP, Adenosine Triphosphate; Mt, Mitochondria; mtDNA, Mitochondrial DNA; mtUPR, mitochondrial unfolded protein response; ROS, Reactive Oxygen Species; AIE, Aggregation-Induced Emission; NIR, Near Infrared; NIR-I, Near-Infrared I; H₂S, Hydrogen sulfide.

**Table 3:** Representative Lysosome-targeted cancer imaging platforms classified by functional motifs.

Probe	Functional motif	Ex/Em (nm)	T/N	Key feature	Limitation	Ref.
OELyso-Gal	Morpholine	610/641	74.6	Enzyme-activated tumor imaging; high signal amplification	β-gal expression heterogeneity; limited penetration	62
NIR-Lys-H_2_S	Morpholine	570-680	1.5	Rapid response; tumor imaging in cells and mice	Limited universality of H₂S as biomarker	63
duNP-DA	DA	780/845	8.2	Dual activation enables detection of small metastases	Complex design and dependence on H₂S expression	64
Lyso-PE	Morpholine	450-550	3.3	Oxidative stress-responsive imaging; tumor-guided surgery	Limited tissue penetration; exogenous ONOO^-^detection	65
SOH	Sulfonium Perchlorate	496/710	2.5	Rapid response; high signal-to-background ratio	Limited tumor specificity; insufficient *in vivo* evaluation	66

Abbreviations: Ex/Em, Ex/Emission; T/N, Tumor-to-Normal ratio; β-gal, β-galactosidase; DA, 2,3-Dimethylmaleic Anhydride; H₂S, Hydrogen sulfide; ONOO-, Peroxynitrite anion.

**Table 4:** Representative ER-targeted tumor imaging platforms based on functional motifs.

Probe	functional motif	Ex/Em (nm)	T/N	Key feature	Limitation	Ref.
BEQ-ER	*p*-Toluenesulfonamide	561/682	2	High ER colocalization; enables ER imaging in living cells and tumor-bearing mice	Dependent on SUR expression; limited deep-tissue imaging capability	79
EICy-P	p-Toluenesulfonamide	680/720	4	Integrates ER targeting with tumor-associated enzyme activation; enables fluorescence imaging-guided PDT	Limited by CYP2J2 expression; lack of clinical validation	80
Te-BOD	Lipophilic moiety	480/565	4	Protein-independent ER targeting; enables discrimination of lung cancer tissues from normal tissues	Relatively slow response; limited tissue penetration	81

Abbreviations: Ex/Em, Ex/Emission; T/N, Tumor-to-Normal ratio; ER, Endoplasmic reticulum; SUR, Sulfonylurea Receptor; CYP2J2, Cytochrome P450, Family 2, Subfamily J, Member 2.

**Table 5:** Representative nucleus-targeted cancer imaging platforms classified by functional motifs.

Probe	functional motif	Ex/Em (nm)	T/N	Key feature	Limitation	Ref.
DSPC-PBP	Protein ligand	545/570	2.1	Tumor-associated protein targeting; enhanced nuclear accumulation	Limited to TYMS-high tumors; insufficient *in vivo* validation	90
1HA4CD	DNA-binding cation	633/700-760	10.6	High signal-to-noise ratio; theranostic application	Nonspecific electrostatic DNA binding	91
CsPbBr₃ PNCs	Antibody	488/525	8.7	Rapid tumor tissue identification; high specificity	Limited nuclear delivery; high antibody cost	92

Abbreviations: Ex/Em, Ex/Emission; T/N, Tumor-to-Normal ratio; TYMS, Thymidylate Synthase.

**Table 6:** Representative GA-targeted tumor imaging platforms based on functional motifs.

Probe	functional motif	Ex/Em (nm)	T/N	Key feature	Limitation	Ref.
TTPI	IMC	480/690	5~6	GA-targeted theranostics; PDT integration	Limited tissue penetration; single-channel fluorescence susceptible to concentration and microenvironment	97
NP-C6-CXB	CXB	OP 488/543 TP 840-	7.9	Cancer cell discrimination; 500 μm tissue imaging; tumor-specific imaging in mice	Short excitation wavelength; solvent-dependent fluorescence	98
GOL-V	Benzene sulfonamide	505-525	3	GA localization; viscosity-responsive imaging; clinical sample imaging	Limited tissue penetration; no NIR/two-photon capability	99
GCSP	Benzene sulfonamide	440/470-496	2.5~3	GA polarity imaging; tumor specimen imaging	Limited penetration; fluorescence interference	100
GA-BOD-S	Benzene sulfonamide	605/681	7~20	Ratiometric NIR imaging; tumor margin visualization; fluorescence-guided surgery	Lack of clinical validation; limited depth evaluation	101
RF-Cou	Furinresponsive peptide	430/475-550		Enzyme-responsive GA imaging; signal amplification	Long response time (6h); insufficient GA targeting validation; limited *in vivo* application	102

Abbreviations: Ex/Em, Ex/Emission; T/N, Tumor-to-Normal ratio; IMC, Indomethacin; CXB, Celecoxib; GA, Golgi apparatus; PDT, Photodynamic Therapy; NIR, Near Infrared.

**Table 7:** Representative organelle-targeted imaging-guided therapeutic systems.

Platform	Imaging role	Organelle event	Therapy	Main limitation	Ref.
Mito-IsTp	Confirms mitochondrial delivery before irradiation	Mitochondrial depolarization and structural damage	Type I/II PDT	Primarily localization evidence; functional feedback is not quantified	120
NO₂/BDP-BT	Hypoxia-activated fluorescence imagin	mitochondrial ROS generation and dysfunction	PDT	limitations include oxygen dependence, tumor heterogeneity, and limited *in vivo* mechanistic validation	121
MPTB	Fluorescence localization and temperature monitoring	Mitochondrial dysfunction; ferroptosis and necroptosis	PDT/PTT	Targeting depends on ΔΨm	122
ADB	Confirms simultaneous mitochondrial and lysosomal localization	Concurrent mitochondrial and lysosomal oxidative damage	PDT	Limited *in vivo* validation and no temporal feedback	22
M@P	Tracks tumor accumulation and supports treatment-time selection	LMP, pyroptosis, ferroptosis and cGAS-STING	PDT/PTT with immune adjuvant	Limited tissue penetration and complex composition	113
PTQ-TPA3	Fluorescence, photoacoustic and temperature monitoring	Lysosomal damage and GSDMD-mediated pyroptosis	Type I PDT/PTT	Limited direct measurement of lysosomal function	116
Ru1	Fluorescence colocalization validates ER delivery	ERs and GSDMD-mediated pyroptosis	PDT	Blue-light excitation; metal-associated toxicity requires evaluation	123
THTTPy	Green-to-red conversion reports activation and relocalization	Lysosome/lipid droplet-to-Mt sequential damage	PDT	Limited hypoxia tolerance and possible spectral interference	115
SgTBB NPs	NIR spatial shift tracks Mt-to-Nucleus migration	Mitochondrial depolarization followed by nuclear DNA damage	PDT	Intratumoral administration and limited immune evaluation	19

Abbreviations: Ex/Em, Ex/Emission; T/N, Tumor-to-Normal ratio; ER, Endoplasmic Reticulum; ERs, Endoplasmic reticulum stress; PDT, Photodynamic Therapy; PTT, Photothermal Therapy; LMP, Lysosomal membrane permeabilization; ICD, Immunogenic Cell Death; GSDMD, Gasdermin D; cGAS-STING, Cyclic GMP-AMP Synthase; ΔΨm, Mitochondrial Membrane Potential; ROS, Reactive Oxygen Species; NIR, Near infrared; NIR-I, near-infrared I.

**Table 8:** Representative NIR-II fluorescence-guided organelle-targeted theranostic platforms.

Platform (Ref.)	Target	Ex/Em (nm)	Imaging information and reported time	Therapy	Key response	limitation
Ru1105 131	Mt, ER and Lysosomes	808/1105	Tumor accumulation and reported irradiation time (48 h)	ROS-mediated phototherapy	Multi-organelle oxidative damage and ICD	Organelle selectivity and long-term safety remain unclear
MPTB 132	Mt → Nucleus	660/1072	Tumor accumulation and treatment timing (24 h); intracellular trafficking	Type I PDT	Mitochondrial depolarization, DNA damage and cGAS-STING activation	Low fluorescence quantum yield; short-wavelength excitation limits deep treatment
CM@ER-Rh NPs 20	ER → Mt	640/1038	NIR-II phosphorescence and treatment timing (24 h)	PDT	ER stress followed by mitochondrial dysfunction	NIR-I excitation; emission at the short edge of NIR-II; limited deep models
LnNP@CUR-RGD 17	Cell membrane → Lysosome	808/1580 imaging; 940 Therapy	Orthogonal NIR-II imaging and treatment timing (6 h)	Curcumin-mediated PDT	Time-dependent uptake and lysosomal delivery	Targeting specificity, energy-transfer efficiency and lanthanide clearance
Indo3 NPs 133	Lysosome	808/1100	Tumor accumulation and treatment timing (12 h)	PDT/PTT	LMP, iron dysregulation and mitochondrial dysfunction	NIR-I excitation; immune response and long-term metabolism insufficiently evaluated
Pt (II) metallacycle 134	Lysosome → Mt	808/1115	Tumor distribution and treatment timing (12 h); reported depth ~7 mm	Type I PDT/chemotherapy	LMP and secondary mitochondrial dysfunction	No specific lysosomal ligand; complex synthesis and Pt-related safety
IR-FCD-Ts NPs 135	ER	1064/1273	Tumor retention and treatment timing (24-48 h)	PDT/PTT with immune activation	ER stress, ICD and T-cell response	Limited ROS generation; evidence primarily from subcutaneous models

Abbreviations: Ex/Em, Ex/Emission; T/N, Tumor-to-Normal ratio; ICD, Immunogenic Cell Death; ER, Endoplasmic reticulum; Mt, Mitochondria; PDT, Photodynamic Therapy; PTT, Photothermal Therapy; NIR-I, Near-Infrared I; NIR-II, Near Infrared II; LMP, Lysosomal membrane permeabilization; ROS, Reactive Oxygen Species; cGAS-STING, Cyclic GMP-AMP Synthase.

## Data Availability

The data supporting the results of this study are available from the corresponding author upon reasonable request.
